# Predicting Intrapartum Acidemia: A Review of Approaches Based on Fetal Heart Rate

**DOI:** 10.3390/bioengineering13020146

**Published:** 2026-01-27

**Authors:** Gabriele Varisco, Giulio Steyde, Elisabetta Peri, Iris Hoogendoorn, Maria G. Signorini, Judith O. E. H. van Laar, Massimo Mischi, Marieke B. van der Hout-van der Jagt

**Affiliations:** 1 Faculty of Electrical Engineering, Eindhoven University of Technology, 5600 MB Eindhoven, The Netherlands; giulio.steyde@polimi.it (G.S.); e.peri@tue.nl (E.P.); j.o.e.h.v.laar@tue.nl (J.O.E.H.v.L.); m.mischi@tue.nl (M.M.); 2 Department of Obstetrics and Gynecology, Máxima Medical Center, 5500 MB Veldhoven, The Netherlands; 3 Eindhoven MedTech Innovation Center (e/MTIC), 5600 MB Eindhoven, The Netherlands; 4 Department of Engineering Sciences, Mälardalen University, 721 23 Västerås, Sweden; 5 Department of Electronics, Information and Bioengineering (DEIB), Politecnico di Milano, 20133 Milano, Italy; mariagabriella.signorini@polimi.it; 6 Department of Science and Medical Innovation, Máxima Medical Center, 5500 MB Veldhoven, The Netherlands; iris.hoogendoorn@mmc.nl; 7 Faculty of Biomedical Engineering, Eindhoven University of Technology, 5600 MB Eindhoven, The Netherlands

**Keywords:** acidemia, fetuses, cardiotocography, fetal heart rate, features

## Abstract

Fetal acidemia, caused by impaired gas exchange between the fetus and the mother, is a leading cause of stillbirth and neurologic complications. Early prediction is therefore essential to guide timely clinical intervention. Several strategies rely on cardiotocography (CTG), which combines fetal heart rate (fHR) with uterine contractions and has led to development of clinical guidelines for CTG interpretation and the introduction of different fHR features. Additionally, ST event analysis, investigating changes in the ST segments of the fetal electrocardiogram (fECG), has been proposed as a complementary tool. This narrative review adopts a systematic approach, with comprehensive searches in Embase and PubMed to ensure full coverage of the available literature, and summarizes findings from 30 studies. Clinical guidelines for CTG interpretation frequently lead to intermediate risk level annotations, leaving the final decision regarding fetal management to clinical experience. In contrast, various fHR features can successfully discriminate between fetuses developing acidemia and healthy controls. Evidence regarding the added value of ST events derived from the scalp electrode remains conflicting, due to concerns about invasiveness. Recent studies on machine learning models highlight their ability to integrate multiple fHR features and improve predictive performance, suggesting a promising direction for enhancing acidemia prediction during labor.

## 1. Background

Fetal acidemia, which can have a respiratory or metabolic origin, originates in the presence of insufficient gas exchange between the fetus and the mother [1,2,3,4]. This deficiency in gas transfer causes a decrease in arterial oxygen concentration, known as hypoxemia, and subsequently in the oxygen level in tissues, known as hypoxia. Respiratory acidemia is identified in the presence of an accumulation of carbon dioxide in the fetal circulatory system and a consequent decrease in blood pH. In the event of a sustained lack of oxygen, energy production can be maintained for a limited amount of time via anaerobic metabolism and the production of lactic acid. Metabolic acidemia, on the other hand, occurs when the amount of bicarbonate delivered by the kidneys is not sufficient to compensate for the amount of lactic acid, which results in an abnormal decrease in blood pH. In case the lack of gas exchange is further prolonged, the fetus can also experience a drop in tissue pH, known as metabolic acidosis. This can lead to perinatal asphyxia, diagnosed in the presence of tissue and organ injury, and ultimately stillbirth, estimated by the World Health Organization at around 900,000 deaths worldwide each year, making it one of the leading causes of perinatal mortality [5,6,7,8]. Additionally, acidemia is also responsible for the occurrence of different neurologic complications leading to motor disabilities and long-term neurodevelopmental impairment, ranging from hypoxic–ischemic encephalopathy to cerebral palsy [2,6,9].

A diagnosis of acidemia is crucial to provide an appropriate and timely response from the clinical team, leading to decisions aimed at reversing the situation (such as changing the maternal position) or expediting delivery to guarantee good perinatal outcomes [3,10]. However, since healthcare professionals rely on a variety of clinical tests and standards to define acidemia, it remains difficult to discriminate between the two origins of acidemia in clinical practice [11,12]. Consequently, the literature is also not consistent, and most studies address acidemia without considering its origin. Following international standards, acidemia is commonly defined using thresholds applied to the umbilical cord artery pH (UA pH) and base deficit (BD) [3]. In particular, the most stringent definition requires both UA pH < 7.0 and BD ≥ 12 mmol/L [3,13], a combination that corresponds to approximately the 0.14th percentile of all births (i.e., about 14 newborns per 10,000 deliveries) [13]. Nevertheless, different studies report different definitions for acidemia, resulting in different reported incidences. A recent review by Olofsson reports an incidence of approximately 7–9% when acidemia is defined as UA pH < 7.20, 1–3% when defined as UA pH < 7.10, and 0.26–1.3% when defined as UA pH < 7.00 [13]. Less stringent thresholds (i.e., higher UA pH or lower BD) therefore classify a larger number of fetuses as acidemic but are generally associated with milder or absent long-term sequelae, whereas more stringent thresholds identify fewer cases with a higher risk of adverse outcomes. Some thresholds frequently reported in research studies (e.g., UA pH < 7.15) can be adopted for methodological reasons, such as obtaining more balanced datasets to train prediction models based on machine learning [14,15], but have limited relevance in clinical practice. Additionally, a variety of thresholds applied to the umbilical cord vein pH (UV pH), BD or negative base excess (BE), and blood lactate concentration derived from an umbilical cord blood gas test, as well as Apgar score, or the need for resuscitation for a predefined time period are also frequently used to define acidemia.

The introduction in the clinical practice of cardiotocography (CTG), which provides tracings for both fetal heart rate (fHR) and uterine contractions, offered clinicians new opportunities to monitor fetal well-being and predict the onset of complications, such as acidemia [16]. According to the American College of Obstetricians and Gynecologists (ACOG), CTG was already employed in 85% of all deliveries in the United States in 2008, monitoring approximately 3.4 million fetuses each year, underscoring its central role in clinical decision-making during labor [17]. Its ability to provide continuous, real-time assessment of fetal heart rate patterns makes it the primary tool for detecting early signs of intrapartum acidemia and guiding timely interventions.

Different strategies were therefore employed in recent years to derive clinical guidelines for CTG interpretation [10,18,19,20,21,22]. Various studies have also investigated the predictive power associated with fHR baseline, deceleration- and acceleration-derived features, which, for instance, include the presence of tachycardia and abnormal fHR patterns [23,24]. The investigation of fetal heart rate variability (fHRV) features allowed for the exploration of the influence of the autonomic nervous system (ANS) on fHR changes [25,26,27,28,29]. In addition to this, the implementation of fetal electrocardiogram (fECG) monitoring strategies in the intrapartum period provided a reliable way to assess changes occurring in the ST segments, defined as ST events [30,31,32,33,34,35,36]. These were introduced to complement other fHR features, a term used throughout this literature review to group fHR baseline, deceleration- and acceleration-derived features, and fHRV features. However, some fECG extraction strategies require invasive fetal scalp electrodes [10,37,38], which limits their routine use compared to the non-invasive monitoring provided by CTG. Newer technologies, including transabdominal fECG, aim to overcome this limitation by offering a non-invasive alternative [39,40,41,42,43]. More recently, machine learning and fHR features were used together to predict the onset of acidemia [44,45,46]. However, it is important to note that despite results derived using several of the previously mentioned strategies (e.g., different fHRV features, machine learning, and fHR features) holding promise for enhancing clinical practice, they have not yet reached full implementation and are therefore primarily used in a research context.

To summarize, there are numerous studies published in the literature that derive information from CTG tracings to predict acidemia, employing heterogeneous approaches. However, there is a lack of an organized overview of the various methods involving clinical guidelines for CTG interpretation, fHR features, and ST events, which makes it difficult to navigate the relevant literature. Therefore, in this literature review, we aim to provide an overview of the most well-established and promising approaches based on these methods for the prediction of acidemia in the intrapartum period, while at the same time highlighting studies that include sufficiently large datasets and have been published within the last 5 years. Although narrative in structure, this literature review follows a systematic approach, including comprehensive searches in Embase and PubMed to ensure a complete overview of the available evidence. As this literature review is based solely on publicly accessible published material, ethical committee approval was not required.

The included studies used a variety of strategies to define the predicted condition as well as its origin (e.g., perinatal asphyxia, respiratory acidemia, metabolic acidemia, metabolic acidosis). Nonetheless, we always used the term acidemia to describe the predicted condition to avoid using non-universally recognized definitions, while at the same time improving the readability and providing more cohesion within this literature review. Nonetheless, precise indications regarding the adopted clinical tests and standards used to define the investigated condition are reported for each included study within the dedicated table. Additionally, we always used the term CTG tracings since it encompasses both fHR and fECG tracings. We also acknowledge that the clinical interpretation of fetal well-being based on fHR is always performed in conjunction with the uterine contraction signal. Further details regarding the literature screening process are presented in Appendix A.

**Figure 1:**
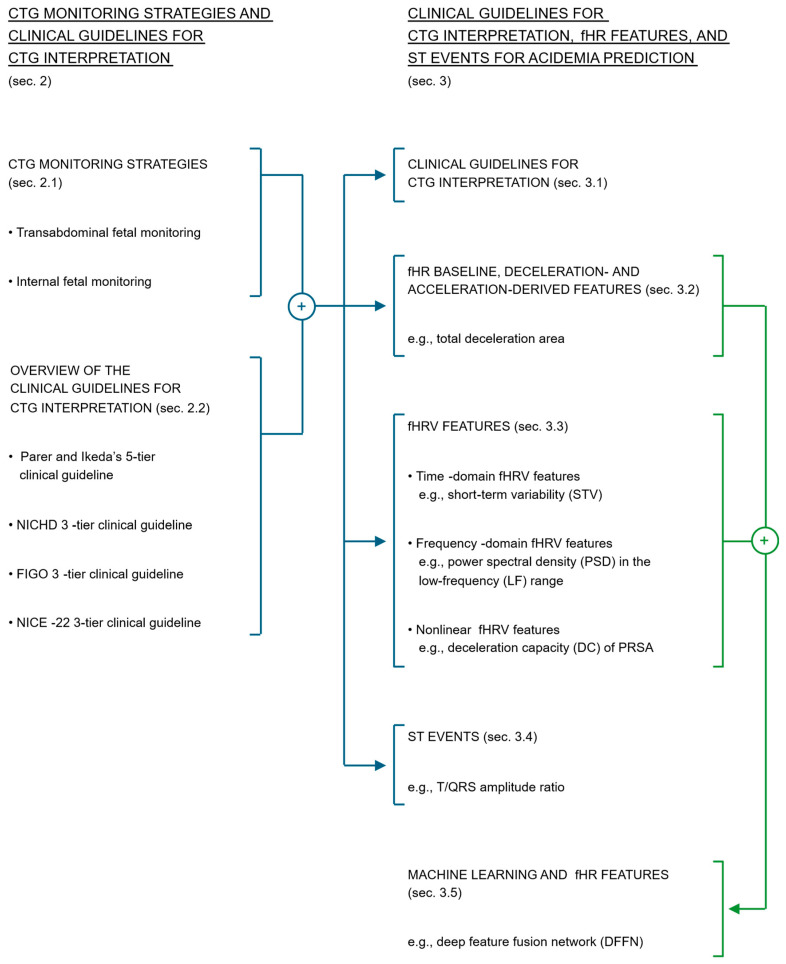
Visual representation of the content of Section 2, cardiotocography (CTG) monitoring strategies and clinical guidelines for CTG interpretation, and Section 3, clinical guidelines for CTG interpretation, fHR features, and ST events for acidemia prediction. Different colors were used to help identify connected sections or sections that were built upon prior knowledge. Acronyms: CTG—cardiotocography, fHR—fetal heart rate, fHRV—fetal heart rate variability.

This literature review is structured as follows. Section 2 presents different CTG monitoring strategies as well as an overview of various clinical guidelines for CTG interpretation. Section 3 presents the included studies that performed a prediction of acidemia using (1) clinical guidelines for CTG interpretation, (2) fHR baseline, deceleration- and acceleration-derived features, (3) fHRV features, and (4) ST events. In addition, prediction models based on (5) machine learning and fHR features are also described. To help the readers navigate through this literature review, a visual representation of the content of these sections and how these relate to each other is also presented in Figure 1. Finally, Section 4 presents the discussion and Section 5 provides an outlook on future directions for improving the prediction of acidemia.

## 2. CTG Monitoring Strategies and Clinical Guidelines for CTG Interpretation

### 2.1. CTG Monitoring Strategies

During the early 1960s, the work of Hon and Lee provided one of the very first systematic descriptions of fHR patterns preceding adverse fetal outcomes, which were primarily obtained by means of invasive fetal electrocardiography and intrauterine pressure measurements [47]. This study therefore highlighted the clinical value of continuous monitoring during labor, thereby laying the foundation for modern CTG, which consists of the continuous synchronous recording of fHR and uterine contractions. Such recordings are typically obtained with a non-invasive transabdominal fetal monitoring technique that simultaneously derives the fHR using a Doppler ultrasound transducer and records uterine contractions using a pressure-sensitive transducer [4,34,48].

The CTG monitor has become the most widely used device to perform fetal monitoring in hospitals all around the world [4,49]. Additionally, its use for continuous intrapartum monitoring became a gold standard for pregnancies characterized by a variety of maternal risk factors and intrauterine conditions, such as suspected fetal growth restriction and preeclampsia [3,17]. However, the visual interpretation of CTG tracings is a subjective process characterized by high inter-observer and intra-observer variability, with low agreement found both when classifying abnormal CTG patterns and when trying to distinguish reassuring CTG tracings from not reassuring [2,17,50]. It has in fact been shown that agreement among clinicians is generally poor when classifying abnormal CTG patterns as well as when assessing whether a tracing is overall reassuring [50]. From the start of CTG monitoring, clinicians and professional societies sought to provide a uniform and coherent framework for interpretation and worked at defining clinical guidelines for CTG interpretation [10,21,22,51] and fHR features [19,28,52,53], in an effort that continues until the present day.

The desire for improved signal quality motivated the development of better sensors for fHR and uterine pressure detection during labor and resulted in the scalp electrode, used in combination with an internal uterine pressure catheter, finding growing use for some decades in clinical practice [17,30,31,37,54,55,56,57]. The scalp electrode comprises a spiral wire attached directly to the fetal presenting part, enabling the acquisition of a high-quality fECG signal. While the electrode facilitates the precise detection of individual heartbeats, the monitoring system employs signal processing to analyze morphological changes in the ST segment (referred to as ST events). Specifically, the system computes an average of multiple consecutive fECG complexes (i.e., most commonly 30) to enhance the signal-to-noise ratio before analysis [30,31,32,33,34,35,36]. The placement of the scalp electrode is limited to third-trimester fetuses (gestational age (GA) ≥ 32 weeks) during labor, provided there is sufficient cervical dilation, and after the rupture of the membranes [10,30,31,38,40]. Different authors also highlighted that due to its invasive nature, it is associated with an increase in neonatal morbidities compared to traditional monitoring techniques [10,37,38]. The trade-off between benefits and risks provided using the scalp electrode compared to and in addition to Doppler ultrasound is, however, still under debate [58,59,60]. Nonetheless, different studies aiming at predicting acidemia were performed by using the latter as well as ST events [36,55,56,59].

In recent years, a lot of attention has also been dedicated to the development of new transabdominal monitoring strategies for CTG based on the principle of electrophysiology, including Nemo Fetal Monitoring System [39,40], Novii Wireless Patch System [41], Femom [42], and Invu [43], which are currently only approved for clinical use in specific regions of the world. Due to their non-invasive nature, these can also be used with intact membranes, hence, also before the onset of labor. In addition to this, electrophysiology-based devices can allow for an investigation of raw fECG and provide an improved quality of the derived fHR [40]. A further description of these devices is, however, outside of the scope of this literature review since they have not been validated yet to predict acidemia.

### 2.2. Overview of the Clinical Guidelines for CTG Interpretation

For a long time, there was little consensus among healthcare professionals regarding CTG patterns indicating signs of acidemia [16]. Consensus regarding the likelihood of onset of this condition was in fact only reached in the presence of recurrent late decelerations, variable decelerations, or significant bradycardia in the absence of fHRV. Therefore, different researchers, healthcare professionals, associations, and societies worked at defining clinical guidelines for CTG interpretation, aiming to reach a more universal consensus about their interpretation and prevent the occurrence of acidemia [61,62]. Table 1 summarizes those that received broader international relevance, transcending national boundaries, and presents their respective categories used to separate CTG tracings, alongside the associated risk for acidemia, the risk of evolution, the required clinical action, as well as additional considerations.

A noticeable effort was made by Parer and Ikeda in 2007, who proposed a 5-tier clinical guideline [16]. These authors identified a total of 134 CTG patterns and annotated them using five color-coded categories (ranging from green to red) to indicate the likelihood of progressing towards more severe patterns associated with higher risks of developing acidemia, following the indications derived from a previous workshop [22]. This was primarily performed by using different thresholds for three fHR features, the baseline fHR, fHRV, and different types of fHR decelerations, with the latter, for instance, being graded based on characteristics such as depth, duration, and nadir [22]. Additionally, the presence of sinusoidal fHR patterns and marked variability were used to support the annotation process.

Following this work, a joint effort between the National Institute of Child Health and Human Development (NICHD), the Society for Maternal-Fetal Medicine (SMFM), and ACOG conveyed an updated interpretation of CTG patterns, which was published in 2008 [17,21]. fHR accelerations were considered together with the fHR features previously identified by Parer and Ikeda while defining the NICHD 3-tier clinical guideline [21]. Category I and III tracings were introduced to indicate normal and abnormal CTG patterns based on their association with acidemia, respectively. Category II tracing allowed instead to describe indeterminate patterns requiring further surveillance and evaluation from healthcare professionals. However, no clear recommendation about fetal management was provided for this tracing.

A revision of the NICHD 3-tier clinical guideline was then presented in 2015 by the International Federation of Gynecology and Obstetrics (FIGO) [10]. The FIGO 3-tier clinical guideline involved the use of the baseline fHR, fHRV, different types of fHR decelerations, fHR accelerations, and uterine contractions. Normal tracing was introduced to indicate an absence of risk of developing acidemia. Pathological tracing, a category introduced to indicate the presence of abnormalities located either in the baseline fHR or fHRV, or presenting repetitive or prolonged decelerations, was used to describe high-risk fetuses requiring immediate clinical action. Finally, suspicious tracing allowed to describe CTG tracings lacking at least one characteristic of the normal ones.

The widely recognized high level of interpretability of the different clinical guidelines for CTG interpretation facilitated their adoption in clinical practice, despite ongoing debate among clinicians about which guideline is superior [61]. National guidelines, such as the SWE-17 3-tier clinical guideline, used in Sweden since 2017, was derived from the (international) FIGO 3-tier clinical guideline and replaced the previously adopted SWE-09 4-tier clinical guideline [18,20]. Recent examples of country-specific revisions also include NICE-17 and NICE-22 3-tier clinical guidelines, issued by the British National Institute for Health and Care Excellence (NICE) in 2017 and 2022, respectively, which updated the recommendations for CTG interpretation in the United Kingdom [20,63,64]. Notably, NICE-22 investigates fHR baseline, fHRV, different types of fHR decelerations, and uterine contractions, alongside the presence of fHR accelerations, to separate normal, suspicious, and pathological tracings. Differences from the FIGO 3-tier clinical guideline include, for instance, the introduction of a specific tachycardia threshold (absent in the FIGO 3-tier clinical guideline) and revised definitions for the type and duration of fHR decelerations, used to classify pathological tracings.

Meanwhile, different international experts gathered in 2018 and 2024, respectively, to develop and refine the first consensus guideline on physiological CTG interpretation, aiming to promote an approach that emphasizes the underlying fetal pathophysiology. While all CTG interpretation strategies have historically been based on physiological principles, this approach places greater focus on identifying the type of hypoxia, compensatory mechanisms, and degree of decompensation for each fetus. These principles have since been adopted in maternity units across several countries [65,66] and led, for instance, to the definition of the CAESARE tool to facilitate their application in clinical practice [67].

It is important to mention that the wide adoption of the aforementioned clinical guidelines for CTG interpretation led to various commercial initiatives to develop automated CTG interpretation systems based on them [19]. Over the years, several commercial devices have been introduced, including SisPorto [56,68,69], STAN by Neoventa [57,70,71], INFANT [72], and Sonicaid FetalCare [73]. All are still in use in clinical practice. Research continues, however, and new prototype devices, such as OxSys [70], have been launched in recent years. These commercial devices incorporate clinical guidelines for CTG interpretation, such as the FIGO 3-tier clinical guideline [56,68,69], as well as different fHR features, such as the short-term variability (STV) and long-term variability (LTV) [55,68,69,73]. Recent iterations of the STAN and SisPorto systems also make use of the scalp electrode to compute ST events [55,68,69]. A further discussion of these commercial devices is outside the scope of this literature review since they have been recently described in more detail [19]. Since different studies investigated the performance of the previously mentioned fHR features and ST events to predict the onset of acidemia, a selection of these studies is presented in Section 3.1, Section 3.3 and Section 3.4 of this literature review.

**Table 1:** Overview of the different clinical guidelines for cardiotocography (CTG) interpretation that received broader international relevance. These include Parer and Ikeda’s 5-tier clinical guideline, NICHD 3-tier clinical guideline, FIGO 3-tier clinical guideline, and NICE-22 3-tier clinical guideline. For each category, the associated risk for acidemia is provided together with the risk of evolution, the required clinical action, and additional considerations. Acronyms: CTG: cardiotocography, fHR—fetal heart rate, fHRV—fetal heart rate variability.

Parer and Ikeda’s 5-tier clinical guideline (2007) [16]	Green	Blue	Yellow	Orange	Red
Risk for acidemia	Absent	Absent	Absent	Borderline/acceptably low	Unacceptably high
Risk for evolution	Very low	Low	Moderate	High	Very high
Action	None	Adopt conservative techniques	Adopt conservative techniques; increase surveillance	Adopt conservative techniques; prepare for urgent delivery	Expedite delivery
Additional considerations	Baseline fHR, fHRV, and different types of fHR decelerations were defined according to the NICHD nomenclature (1997) [22]				
NICHD 3-tier clinical guideline (2008) [17,21]	Category I		Category II	Category III	
Risk for acidemia	Absent		Absent/acceptably low	High	
Risk for evolution	Very low		Low/high	High	
Action	None		Perform evaluation; continue surveillance; and perform re-evaluation	Perform prompt evaluation; expedite delivery	
Additional considerations	Baseline fHR, fHRV, different types of fHR decelerations, fHR accelerations, and uterine contractions were defined according to Macones et al. (2008) [21]				
FIGO 3-tier clinical guideline (2015) [10]	Normal		Suspicious	Pathological	
Risk for acidemia	Absent		Low	High	
Risk for evolution	Very low		Low/high	High	
Action	None		Correct reversible causes if identified; perform close monitoring; use additional methods to evaluate fetal oxygenation	Adopt immediate action to correct reversible causes; adopt additional methods to evaluate fetal oxygenation; expedite delivery	
Additional considerations	Baseline fHR, fHRV, different types of fHR decelerations, fHR accelerations, and uterine contractions were defined according to Ayres-de-Campos et al. (2015) [10]				
NICE-22 3-tier clinical guideline (2022) [64]	Normal		Suspicious	Pathological	
Risk for acidemia	Absent		Moderate	High	
Risk for evolution	Very low		Low/high	High	
Action	None		Perform evaluation (considering whether fHR accelerations are identified); perform full risk assessment; expedite delivery	Perform full risk assessment; expedite delivery	
Additional considerations	Baseline fHR, fHRV, different types of fHR decelerations, fHR accelerations, and uterine contractions were defined according to the NICE nomenclature (2022) [65]				

## 3. Clinical Guidelines for CTG Interpretation, fHR Features, and ST Events for Acidemia Prediction

### 3.1. Clinical Guidelines for CTG Interpretation

The clinical guidelines for CTG interpretation presented in Section 2.2. represent the gold standard in clinical practice [10,17,61]. Different studies tried to identify which clinical guideline for CTG interpretation can better predict acidemia [20,74]. A selection of these studies is presented in this section and summarized in Table 2, following the order in which the different clinical guidelines for CTG interpretation were introduced in Section 2.2.

Some limitations were highlighted for Parer and Ikeda’s 5-tier clinical guideline by Elliott et al., who aimed at standardizing the annotation process of CTG tracings using color-coded categories and a dedicated software [75]. They investigated CTG tracings from the last 3 h preceding the delivery in 60 acidemic fetuses developing neonatal encephalopathy (defined as UA BD > 12 mmol/L), 280 acidemic fetuses without neurologic complications, and 2132 controls. Only 8.3% of the group with neonatal encephalopathy received a red annotation, and this only lasted 5.2 min on average. Furthermore, the authors investigated the minimum amount of time spent above a specific color-coded category that could discriminate between groups of fetuses and computed the receiver operating characteristic (ROC) curves. In particular, the highest AUROC, equal to 0.83, was obtained when grouping all color categories above blue in the classification of acidemic fetuses developing neonatal encephalopathy vs. healthy controls. Smaller differences were found between acidemic fetuses that did not develop encephalopathy and controls, and the ROC curve for this comparison was not reported. Critically, this separation has limited clinical distinctive power for identifying or stratifying significant acidemia, as blue represents the lowest-risk classification. More clinically relevant thresholds yielded lower performance. Separating yellow or higher resulted in an AUROC equal to 0.77, while progressively higher thresholds were associated with further reductions in discriminative ability. These findings indicate that although standardized annotation improves reproducibility, color-based thresholding alone provides limited diagnostic performances.

One of the main criticisms of the NICHD 3-tier clinical guideline is related to the fact that up to 85% of CTG tracings occurring in daily practice can fall within category II, leaving the decisions about fetal management to the medical team in charge and therefore leading to different perinatal outcomes [74,76,77]. Clark et al. tried to face this problem by using a tree-based algorithm for fetal management followed by a clinician’s decision and compared it with standardized decisions adopted in clinical practice [78]. Both approaches were considered while investigating 120 acidemic fetuses (defined as UA BD > 12 mmol/L) and 120 matched controls (defined as UA BD < 8 mmol/L), selected as the nearest eligible deliveries matched for parity. Algorithm performance was assessed under the assumption of a 30 min evaluation period for noncritical category II CTG tracings, followed by delivery within 30 min. The tree-based algorithm followed by a decision from healthcare professionals led to a higher number of operative intervention decisions for the acidemic group compared to standardized clinical practice (55 vs. 36) and was identified as the best-performing method. Additionally, it enabled earlier recognition of fHR patterns associated with metabolic acidemia, an advantage that is frequently overlooked in most research studies which instead only focus on reporting results for predictive performance. However, the overall performance gain was deemed as modest, with only about half of acidemic infants potentially identifiable even under idealized conditions, which the authors noted is unlikely to reflect routine clinical practice. An important limitation of the NICHD 3-tier clinical guideline was pinpointed by Bruno et al. while investigating 4274 fetuses, including 42 developing acidemia at birth (defined as UA pH ≤ 7.1) [79]. A considerable number of cases (i.e., 13 fetuses, or 31%) had their CTG tracings from the 30 min preceding the delivery assigned to category I.

A comparison of the performance of Parer and Ikeda’s 5-tier and NICHD 3-tier clinical guidelines was conducted by Coletta et al. in 2012 while considering the last 30 to 60 min of CTG tracings from 24 acidemic fetuses (defined as UA pH < 7.0) and 24 matched controls [74]. This study showed higher sensitivity and the same specificity for the former clinical guideline for CTG interpretation while predicting acidemia (i.e., SE = 79.2% found with orange or red tracings vs. 12.5% found with category III, SP = 100% vs. 100%). Nonetheless, the limited number of included CTG tracings and a single reviewer responsible for performing both annotations represent important limitations that should be tackled in future studies.

More recently, Kling et al. evaluated the performance of the FIGO-15 3-tier, SWE-09 4-tier, SWE-15 3-tier, NICE-17 3-tier, and NICE-22 3-tier clinical guidelines in predicting the onset of acidemia [20]. These authors included CTG tracings from the last 1 h before birth which were derived from a total of 573 subjects, with annotations performed independently by two reviewers and disagreements resolved by a third. Acidemia was defined as UA pH < 7.1, resulting in 38 cases. A key result from this study was that NICE-22 3-tier and SWE-09 4-tier clinical guidelines achieved the highest sensitivity for pathological tracings (both 92%; 95% CI 79–98%). On the other hand, the highest specificity for pathological tracings was instead found for the SWE-17 3-tier (91%; 88–93%) and FIGO-15 3-tier clinical guidelines (90%; 88–93%). Based primarily on its superior sensitivity, the authors identified the NICE-22 3-tier clinical guideline as the safest to predict acidemia. As in previous studies, however, a major limitation was the small number of acidemia cases included.

Some studies were also used to describe the predictive ability of the different clinical guidelines for CTG interpretation when associated with additional parameters. Pruksanusak et al., who investigated the last 2 h of CTG tracings from 36 fetuses developing acidemia (identified using multiple strategies including UA pH < 7.1 or BD ≥ 12 mmol/L within 60 min of birth) and 679 controls, compared all three clinical guidelines for fHR interpretation as well as different risk factors [80]. They showed that category II and suspicious tracings, as defined in the NICHD and FIGO 3-tier clinical guidelines, respectively, can be characterized by higher sensitivity and lower specificity in predicting acidemia compared to yellow and orange tracings from Parer and Ikeda’s 5-tier clinical guideline (SE = 67.0% vs. 61.0% vs. 36.0% vs. 8.0%, SP = 65.0% vs. 47.0% vs. 85.0% vs. 99.0%). On the other hand, the inclusion of maternal risk factors slightly increased the performance of Parer and Ikeda’s 5-tier clinical guideline compared to the NICHD and FIGO 3-tier clinical guidelines, as shown by the increased AUROC value (0.72 vs. 0.68 vs. 0.63).

To summarize, as indicated by the studies included in this section, the different clinical guidelines for CTG interpretation have been frequently used in clinical practice to stratify CTG tracings according to the risk of developing fetal complications over the years [10,21,22]. Results reported by different authors revealed an inherent limitation of the different clinical guidelines for fHR interpretation: their use often leads to intermediate risk level annotations for most CTG tracings, which leaves decisions regarding consequent fetal management to the expertise developed by each medical team [74,76,77]. Since this might vary significantly, the use of clinical guidelines for CTG interpretation can lead to very different perinatal outcomes. In addition to this, it remains unclear which clinical guideline for CTG interpretation provides better support to healthcare professionals interested in predicting acidemia. Studies that focused on their comparison reported contrasting results for the sensitivity and specificity metrics [74,80].

**Table 2:** Overview of studies using clinical guidelines for cardiotocography (CTG) interpretation to predict acidemia.

Study	Population and CTG Tracings	Methods	Key Results	Distinct Limitations
Elliott et al. (2010) [75]	• 2472 total fetuses (GA > 35.0 weeks) including the following: - 60 with acidemia and developing neonatal encephalopathy (UA BD > 12 mmol/L) - 280 with acidemia without neurologic complications (UA BD > 12 mmol/L) - 2132 controls • Use of CTG tracings from the last 3 h before delivery	• Use of a dedicated software to annotate CTG tracings from three groups of fetuses according to Parer and Ikeda’s 5-tier clinical guideline • This software uses proprietary signal processing techniques and pattern recognition algorithms • Use of metrics (e.g., ROC curves and AUROC values)	• Only 8.3% of the fetuses developing acidemia and neonatal encephalopathy ever reached the red annotation in the last 3 h preceding the delivery, and this happened on average only for 5.2 min • An AUROC value equal to 0.77 was found when investigating the minimum time spent above a specific color-coded category, using yellow as the threshold, to discriminate between fetuses developing neonatal encephalopathy and controls	• Clinicians had access to the included CTG tracings and might have influenced clinical management
Clark et al. (2017) [78]	• 240 total fetuses including the following: - 120 with acidemia (mean GA = 39.9 weeks, UA BD > 12 mmol/L) - 120 matched controls (mean GA = 39.9 weeks, UA BD < 8 mmol/L) • Use of 30-min noncritical category II CTG tracings followed by delivery within 30 min	• Compared a tree-based algorithm for fetal management followed by a decision from healthcare professionals with standardized decisions adopted in clinical practice consisting of the use of the NICHD 3-tier clinical guideline • Use of statistical tests (e.g., chi-squared and Fisher’s exact test)	• The use of a tree-based algorithm followed by a decision from healthcare professionals led to a higher number of operative intervention decisions for fetuses developing acidemia compared to standardized clinical performance (55 vs. 36) and enabled earlier recognition of fHR patterns associated with metabolic acidemia	• The analysis was restricted to a specific set of fetuses, characterized by a 30 min noncritical category II CTG tracings followed by delivery within 30 min
Bruno et al. (2020) [79]	• 4274 total fetuses including the following: - 42 with acidemia (mean GA = 39.2 weeks, UA pH ≤ 7.1) - 4232 controls (mean GA = 38.9 weeks) • Use of CTG tracings from the last 60 min before delivery	• Verify whether assigning Category I of the NICHD 3-tier clinical guideline to CTG tracings from the 60 min preceding delivery guarantees absence of acidemia. • Use of statistical tests and metrics (e.g., sensitivity and specificity)	• A considerable number of cases (i.e., 13 fetuses, or 31%) had category I assigned to CTG tracings from the 30 min preceding the delivery	• Limited number of included CTG tracings
Coletta et al. (2012) [74]	• 48 total fetuses including the following: - 24 with acidemia (mean GA = 38.7 weeks, UA pH < 7.0) - 24 matched controls (mean GA = 38.8 weeks) • Use of CTG tracings from the last 30 to 60 min before delivery	• Comparison between Parer and Ikeda’s 5-tier and NICHD 3-tier clinical guidelines for the task of predicting acidemia • Use of statistical tests and metrics (sensitivity and specificity)	• Parer and Ikeda’s 5-tier clinical guideline showed higher sensitivity and the same specificity compared to the NICHD 3-tier clinical guideline while predicting acidemia (i.e., SE = 79.2%, found with orange or red tracings, vs. 12.5%, found with category III, SP = 100% vs. 100%)	• Limited number of included CTG tracings • A single reviewer was responsible for performing annotations considering both clinical guidelines
Kling et al. (2024) [20]	• 573 total fetuses including the following: - 38 with acidemia (GA = 34–36.6 weeks, UA pH < 7.1) - 535 controls (GA = 34–36.6 weeks, UA pH ≥ 7.1) • Use of CTG tracings from the last 1 h before birth	• Comparison of FIGO-15 3-tier, SWE-09 4-tier, SWE-15 3-tier, NICE-17 3-tier, and NICE-22 3-tier clinical guidelines in their ability to predict the onset of acidemia • Use of statistical tests and metrics (sensitivity and specificity)	• The highest sensitivity for pathological tracings was found for NICE-22 3-tier and SWE-09 4-tier clinical guidelines (both 92%; 95% CI 79–98%) • The highest specificity for pathological tracings was found for SWE-17 3-tier (91%; 88–93%) and FIGO-15 3-tier clinical guidelines (90%; 88–93%)	• Limited number of included CTG tracings
Pruksanusak et al. (2017) [80]	• 715 total fetuses including the following: - 36 with acidemia (mean GA = 38.8 weeks, UA pH < 7.1 or BD ≥ 12 mmol/L within 60 min of birth or other strategies) - 679 controls (mean GA = 39.0 weeks) • Use of CTG tracings from the last 2 h before delivery	• Comparison of the predictive power for acidemia of the different clinical guidelines for CTG interpretation • Investigation as to whether this predictive power can be improved using additional maternal- associated risk factors • Use of statistical tests and prediction models (e.g., Pearson’s chi-squared and logistic regression)	• Category II and suspicious tracings can be characterized by higher sensitivity and lower specificity in predicting acidemia compared to yellow and orange tracings (SE = 67.0% vs. 61.0% vs. 36.0% vs. 8.0%, SP = 65.0% vs. 47.0% vs. 85.0% vs. 99.0%) • The inclusion of maternal risk factors slightly increased the performance of Parer and Ikeda’s 5-tier clinical guideline, which outperformed both the NICHD 3-tier and FIGO 3-tier clinical guideline as shown by the increased AUROC value (0.72 vs. 0.68 vs. 0.63)	• Very limited number of included fetuses developing acidemia • Metrics derived from the use of prediction models combining clinical guidelines for CTG interpretation, and additional risk factors were not computed

### 3.2. fHR Baseline, Deceleration-, and Acceleration-Derived Features

Even before the introduction of the clinical guidelines for CTG interpretation, different research groups investigated whether different fHR baseline, deceleration-, and acceleration-derived features were able to predict acidemia [81,82,83]. Over time, fHR baseline and deceleration-derived features have shown more promising results than fHR acceleration-derived features, a reason why they are still extensively frequently investigated in present day [23,24,77,84,85,86,87,88]. Therefore, a selection of these studies is presented in this section and summarized in Table 3.

Several studies highlighted the potential predictive power associated with changes in the fHR baseline. In particular, it was pointed out by Cahill et al. that tachycardia (i.e., baseline fHR > 160 bpm) holds a significant association with acidemia and performs better in this task compared to several other fHR features derived from the NICHD 3-tier clinical guideline [24]. Tachycardia showed a high specificity together with very low sensitivity and precision (SP = 95.7%, SE = 14%, PRE = 3.4%). It is, however, important to notice that some of the obtained results (e.g., PRE) were influenced by the severe imbalance that characterized the population included in this study, which consisted of 5331 controls and 57 fetuses developing acidemia (defined as UA pH < 7.1). On the other hand, in line with other studies [77], bradycardia (i.e., baseline fHR < 110 bpm) was never found in acidemic fetuses and occurred very rarely in controls. These results indicate that further research is needed to investigate the predictive power of fHR baseline, while considering bigger and more balanced populations.

The term saltatory pattern was first introduced with the FIGO 3-tier clinical guideline to define abrupt “up and down” fluctuations in the baseline fHR characterized by an amplitude > 25 beats/min and a duration > 30 min [23,84]. Different studies reported that saltatory patterns are very rarely observed in CTG tracings and are therefore considered difficult to use to predict fetal acidemia [10,89]. However, Gracia-Perez-Bonfils et al. investigated a total of 484 CTG tracings and identified that short saltatory patterns lasting more than 1 min and more than 2 min, often referred to as zigzag patterns, were significantly more likely to be observed in fetuses with 7.0 < UA pH ≤ 7.1 and 7.1 < UA pH ≤ 7.2 compared to controls [23]. An important limitation of this study was, however, the fact that UA pH ≤ 7.0 was found in only three of the included fetuses and therefore did not allow meaningful results to be reached for fetuses characterized by the most critical conditions. A significant association between short saltatory patterns lasting more than 2 min, acidemia, and other severe complications (identified, for instance, by using UA pH < 7.1 and/or BE < −12.0 mEq/L) was also identified by Tarvonen et al. while considering more extensive populations (160 fetuses with severe complications, 1208 with moderate complications, and 3620 controls) [84]. The authors also managed to identify an interesting pattern that had not been previously described, which was localized within the last 2 h before birth and was limited to fetuses with severe complications. This pattern involved a consistent decline in the occurrence of short saltatory patterns, combined with a rise in the frequency of late decelerations up to 1 h before delivery, followed by a subsequent decrease. This information could be useful when developing future prediction models that can process temporal information, such as hidden Markov models or long short-term memory (LSTM) networks. It is also important to notice that the scalp electrode was used for monitoring most of the included fetuses (91.4%). Future studies should focus on verifying whether these results can also be achieved with a non-invasive approach.

Since the first investigations of different types of fHR decelerations were performed, it became clear that they are associated with a significant decrease in UA pH [88,90]. Some follow-up studies, which also include invasive animal studies as well as mathematical simulations, confirmed that late decelerations, which typically follow uterine contractions and are characterized by at least 30 s of decrease in fHR from their onset to the nadir, are a consequence of uteroplacental insufficiency and tend to progress towards acidemia [62,84,90,91]. Variable decelerations, characterized by a variable duration and onset with respect to uterine contractions, were instead identified in the presence of umbilical cord or head compression, as the transient time to reduced fHR is related to a local interruption in fetal blood flow [92,93]. Recently, Fogelberg et al. systematically investigated the relation between different types of decelerations and their association with acidemia in a large cohort of fetuses, consisting of 365 cases and 730 controls [94]. These authors identified a strong association between acidemia and late or prolonged decelerations, a moderate association with combined decelerations, and little to no association between variable decelerations and acidosis. These results further motivated investigations of the correlation between deceleration-derived features and a decrease in UA pH.

An important contribution to this research was provided with the introduction of the total deceleration area, whose computation does not require separating different types of decelerations [86]. Total deceleration area allows to combine information regarding the length, depth, and frequency of the decelerations, reflecting the severity of baroreceptor, chemoreceptor, and/or catecholamine trigger (i.e., in response to a blood pressure change and hypoxemia) [93]. Total deceleration area showed significant differences when comparing fetuses developing acidemia and controls in different studies [24,85]. Significant differences for total deceleration area as well as for the other fHR deceleration-derived features (e.g., number of severe decelerations with a nadir ≤ 60 bpm, number of decelerations > 60 s) were also identified by Gamboa et al. while comparing 102 fetuses developing acidemia (defined as UA pH ≤ 7.1 and BD > 8 mmol/L) and 102 controls [77]. More recently, Furukawa et al. used total deceleration area together with the counts of decelerations to investigate changes over time in the last 5 h before delivery using 30 min CTG tracings. These authors compared 132 fetuses developing acidemia (defined as UA BD > 12 mmol/L) with 1498 controls [86]. Both total deceleration area and the count of variable decelerations were found to be significantly different only in the last 2 h, thus providing an important indication about when the characteristics of the two groups might start diverging.

An interesting approach named fHR fragmentation was recently developed by Costa et al. to identify acidemic fetuses [95,96]. They used the CTU-UHB dataset, which is publicly available at PhysioNet and includes a total of 552 CTG tracings from the 90 min before delivery [97], either derived using Doppler ultrasound or a scalp electrode, as well as UA blood gas data (including the pH and pO_2_) [98]. The authors defined different fHR fragmentation features such as the percentage of inflection points in the fHR (PIP, which indicates changes in the sign of the derivative), the inverse average length of acceleration and deceleration segments (IALS), and the percentage of short accelerations or deceleration segments (PSS). A peculiarity of fHR fragmentation features, compared to total deceleration area, is that they only consider the length of accelerations and decelerations while ignoring their magnitude, a characteristic which further simplifies their computation. PIP, IALS, and PSS showed significant differences when comparing 39 fetuses developing acidemia (defined as UA pH ≤ 7.15) with 207 controls. Fewer differences, however, were noticed when the CTG tracings were downsampled to 2 Hz, showing an important limitation that characterizes fHR fragmentation features.

**Table 3:** Overview of studies using fetal heart rate (fHR) baseline, deceleration-, and acceleration-derived features to predict acidemia.

Study	Population and CTG tracings	Methods	Key results	Distinct limitations
Cahill et al. (2012) [24]	• 5388 total fetuses including the following: - 57 with acidemia (mean GA = 39.0 weeks, UA pH < 7.1) - 5331 controls (mean GA = 39.0 weeks) • Use of CTG tracings from the last 30 min before delivery	• Comparison of fHR features derived from the NICHD 3-tier clinical guideline and fHR deceleration-derived features, including total deceleration area based on their power to predict acidemia • Use of statistical tests, metrics, and prediction models (e.g., Student’s *t*-test and backward stepwise logistic regression)	• Tachycardia held a significant association with acidemia and performed better than other fHR features derived from the NICHD 3-tier clinical guideline (SP = 95.7%, SE = 14%, PRE = 3.4%) • Bradycardia occurred rarely in the controls and did not occur at all in fetuses developing acidemia • Total deceleration area was significantly associated with acidemia after adjusting for labor and delivery conditions	• The included population was characterized by a severe imbalance
Gracia-Perez-Bonfils et al. (2021) [23]	• 484 total CTG tracings (GA range = 37.0–42.0 weeks) including the following: - 3 with UA pH ≤ 7.0 - 18 with 7.0 < UA pH ≤ 7.1 - 73 with 7.1 < UA pH ≤ 7.2 - other CTG tracings split based on UV pH or considered as controls • Use of CTG tracings lasting ≥ 30 min during labor	• Investigation of the incidence of the saltatory patterns and their correlation with perinatal outcomes, including acidemia • Use of a statistical test (i.e., chi-squared test with odds ratios)	• Short saltatory patterns lasting ≥ 1 min and ≥ 2 min were significantly more likely to be observed in fetuses with 7.0 < UA pH ≤ 7.1 and 7.1 < UA pH ≤ 7.2 compared to controls	• Only a limited number of fetuses with UA pH ≤ 7.0 was included
Gamboa et al. (2017) [77]	• 204 total fetuses including the following: - 102 with acidemia (mean GA = 279.5 days, UA pH ≤ 7.1 and BD > 8 mmol/L) - 102 controls (mean GA = 278.6 days) • Use of CTG tracings from the last 30 min before delivery	• Comparison of fHR features derived from the NICHD 3-tier clinical guideline and total deceleration area based on their power to predict acidemia • Use of statistical tests, metrics and prediction models (e.g., paired Student’s *t*-test and backward stepwise logistic regression)	• Bradycardia was hardly observed • Total deceleration area, number of severe decelerations with a nadir ≤ 60 bpm, number of decelerations > 60 s were significantly higher in fetuses developing acidemia	• Only a limited number of fetuses was included • Total deceleration area was calculated manually
Tarvonen et al. (2021) [84]	• 4988 total fetuses including the following: - 160 with severe complications (mean GA = 40.5 weeks, UA pH < 7.10 and/or BE < −12.0 mEq/L and/or other strategies) - 1208 with moderate complications (mean GA = 40.2 weeks) - 3620 controls (mean GA = 40.2 weeks) • Use of CTG tracings from the last 2 h before delivery	• Investigation of possible associations between fHR baseline, deceleration-, and acceleration- derived features, including short saltatory patterns and acidemia • Use of statistical tests and prediction models (e.g., independent samples *t*-test and logistic regression)	• Short saltatory patterns lasting > 2 min were significantly associated with acidemia and severe complications • In fetuses with severe complications, the frequency of short saltatory patterns decreased continuously in the 2 h before delivery, whereas late decelerations increased up to 1 h before birth and then decreased again	• CTG tracings were recorded using a scalp electrode for 91.4% of fetuses
Furukawa et al. (2019) [86]	• 1630 total fetuses including the following: - 132 with acidemia (mean GA = 40.0 weeks, UA BD > 12 mmol/L) - 1498 controls (mean GA = 39.6 weeks) • Use of CTG tracings from the last 5 h before delivery	• Investigation of the predictive power of fHR deceleration- derived features from hours close to delivery for acidemia • Use of statistical tests and metrics (e.g., ROC curves and AUROC values)	• Total deceleration area and counts of variable decelerations showed significantly higher values in fetuses developing acidemia only in the last 2 h • Total deceleration area showed the highest discrimination power based on its AUROC value equal to 0.70 compared to other fHR deceleration-derived features	• No further analysis of metrics following the computation of AUROC values was performed
Costa et al. (2021) [96]	• 246 total CTG tracings from the CTU-UHB dataset including the following: - 39 with acidemia (mean GA = 40.0 weeks, UA pH < 7.15) - 207 controls (mean GA = 39.0 weeks) • Use of CTG tracings from the last 4 h before delivery	• Introduction of different fHR fragmentation features • Investigation of their predictive power for acidemia and comparison with traditional fHR features derived from the FIGO 3-tier clinical guideline • Use of statistical tests (e.g., Mann–Whitney U-test and Cliff’s delta)	• PIP, IALS, and PSS values were significantly lower in fetuses developing acidemia • Lower prediction power was identified when using fHR fragmentation features computed using CTG tracings downsampled to 2 Hz	• The sampling frequency of the CTG tracings affects results obtained with all fHR fragmentation features • Only a limited number of fetuses developing acidemia was included

To summarize, different fHR baseline and deceleration-derived features reported in this section showed a significant correlation with acidemia, proving that even fHR features characterized by a simple computation can contribute to the prediction of this condition. The most promising approaches include the use of both traditional fHR features, such as total deceleration area [24,77,85,86], as well as novel ones, such as those related to fHR fragmentation [96]. The use of short saltatory patterns and late decelerations is also considered promising to predict the onset of acidemia and other severe complications since changes occurring in the last 2 h were presented in detail for both cases and controls [84]. Therefore, if these results are confirmed in future studies, these fHR baseline and deceleration-derived features could be helpful to provide a timely interpretation of changes occurring to the fHR before the onset of acidemia.

### 3.3. fHRV Features

A variety of fHRV features, able to describe variability with respect to the fHR baseline, have been used to predict acidemia. These are commonly divided into time-domain, frequency-domain, and non-linear fHRV features and are presented in this section following this order. An overview of the included studies is summarized in Table 4, whereas further details regarding their computation can be found both in their original studies, as well as in dedicated literature reviews [28,52,53,99].

#### 3.3.1. Time-Domain fHRV Features

Over the past 30 years, several studies investigated the ability of two time-domain fHRV features, short-term variability (STV) and long-term variability (LTV), to predict acidemia [25,96,100,101,102,103,104]. STV is defined as the mean fHR difference between successive 3.75 s RR-intervals segments found in 1 min periods [25,100,102,103]. LTV is defined as the difference between the minimum and maximum value of the mean fHR found in 1 min periods [100,102]. As the STV can provide information regarding high-frequency variability, it has been hypothesized that this feature provides a way to investigate the modulation of the fHR provided by the parasympathetic nervous system (PNS) [96,102]. LTV has instead been related to the modulation of both the sympathetic nervous system (SNS) and the PNS [96,104].

Schiermeier et al. found no significant difference in STV when investigating the last 30 min of CTG tracings before fetal scalp blood sampling (FBS) in 197 fetuses, regardless of the measured fetal scalp pH levels [25]. This study was, however, characterized by the important limitation that only nine acidemic fetuses with a fetal scalp pH ≤ 7.2 were included. More recently, Lu et al. investigated changes in the STV in the intrapartum period by considering up to 120 min before each FBS in a total of 1070 fetuses having even multiple FBS [103]. CTG tracings were separated and consisted, depending on the 30 min period that was considered, of up to 114 for the acidemic group (defined as 4.8 mmol/L< FBS ≤ 6.6 mmol/L) and 1011 for the control group (FBS < 4.2 mmol/L). Significantly higher mean and median STV values were always found in the acidemic group, a result that suggests that this fHRV feature can be used to predict acidemia at least up to 120 min before its onset.

**Table 4:** Overview of studies using fetal heart rate variability (fHRV) features to predict acidemia.

Study	Population and CTG Tracings	Methods	Key Results	Distinct Limitations
Lu et al. (2018) [103]	• 1070 total fetuses (median GA = 40.0 weeks + 3.0 days) which received 2134 fetal scalp blood sampling (FBS). This resulted in the inclusion of different groups of CTG tracings including up to: - 114 with acidemia (4.8 mmol/L < FBS ≤ 6.6 mmol/L) - 1011 controls (FBS < 4.2 mmol/L) • Use of CTG tracings from the last 120 min before FBS	• Investigation of the contribution of short-term variability (STV) in predicting the onset of acidemia in the 120 min preceding FBS • Separation of CTG tracings into 30-min periods • Use of statistical tests and metrics (e.g., Mann–Whitney U-test and Spearman’s Rank test)	• Significantly higher mean and median STV values were found in the acidemic group in each 30 min periods • Weak growing trends for the STV were found over time, progressing towards FBS for acidemic fetuses compared to control	• A population of high-risk fetuses, all of whom presented CTG changes indicating FBS, was included • STV was calculated using a novel modified algorithm, which had only been validated in another study from the same group of authors
Gatellier et al. (2021) [102]	439 total CTG tracings from the CTU-UHB dataset including the following: - 43 with acidemia (mean GA = 39.9 weeks, UA pH ≤ 7.10) - 396 controls (mean GA = 40.0 weeks) • Use of CTG tracings from the last 90 min before delivery	• Investigation of the contribution of different time-domain fHRV features, including the STV, long-term variability (LTV) and Fetal Stress Index (FSI) in the prediction of acidemia • Use of statistical tests and metrics (e.g., Student’s *t*-test)	• Higher values for different fHRV features, including the STV, were found in fetuses developing acidemia in 5 min intrapartum periods characterized by maximum Fetal Stress Index value (FSI_max_) • Statistical significance was only found when considering the latter period	• A visual selection was performed to include the 5 min periods characterized by stable fHR • Such long stable periods, characterized by the absence of accelerations and decelerations, cannot always be found using intrapartum CTG tracings
Butruille et al. (2017) [105]	• 299 total CTG tracings (GA = 36.0–42.0 weeks) including the following: - 12 with acidemia (UA pH < threshold according to the different GA) - 287 controls • Use of CTG tracings from the last 30 min before delivery	• Investigation of the contribution of the FSI and derived statistics in the prediction of acidemia • Use of statistical tests (e.g., Mann–Whitney U-test and chi-squared test)	• Significantly lower values for the FSI_min_ and FSI_mean_ were found in fetuses developing acidemia, indicating mitigation of the whole response of the ANS caused by a prolonged hypoxic condition	• Only a limited number of CTG tracings leading to acidemia was included
Van Laar et al. (2008) [26]	• 458 total fetuses (GA ≥ 20.0 weeks) from six studies • Use of different definitions for acidemia (e.g., UA pH < 7.05 or UA pH < 7.2) • Use of both antepartum and intrapartum CTG tracings	• Systematic review aiming at verifying whether a correlation exists between frequency-domain fHRV features and acidemia while considering different stages of pregnancy	• Most studies investigating intrapartum CTG tracings reported a general decrease in the PSD in the LF range in fetuses developing acidemia • One study that used the highest threshold to discriminate acidemic fetuses from controls (i.e., UA pH < 7.2) reported a decrease in the PSD in the LF range in fetuses developing acidemia • One study reported an initial increase in PSD in the LF range and in the LF/HF ratio followed by a decrease closer to birth only for the former fHRV features in acidemic fetuses	• Use of different definitions of acidemia, options to separate the spectral bands, and use of CTG tracings derived using Doppler ultrasound or a scalp electrode
Van Laar et al. (2010) [71]	• 20 total fetuses including the following: - 10 with acidemia (GA = 283 days, UA pH < 7.05) - 10 controls (GA = 278 days) • Use of CTG tracings from the last 3 h before delivery	• Investigation of the correlation between frequency-domain fHRV features computed using both absolute and relative values with acidemia • Use of statistical tests (e.g., Student’s *t*-test and ANOVA)	• PSD in the LF and HF ranges using relative values were found to be statistically significant and useful to discriminate fetuses developing acidemia from controls only when considering the last 30 min before delivery • Higher values for the relative LF were found in fetuses developing acidemia whereas higher values for the relative HF were found in controls	• Only a limited number of fetuses was included
Castro et al. (2021) [28]	• 246 total CTG tracings (median GA = 40.0 weeks) from the CTU-UHB dataset leading or not to acidemia based on the different thresholds (i.e., UA pH ranging from 7.05 to 7.2) • Use of CTG tracings from the last 35 min included in the CTU-UHB dataset	• Systematic review focused on collecting frequency bands and relative ranges which were used in previous studies to perform intrapartum fHR spectral analysis • Evaluation of their performance when these were used to discriminate CTG tracings leading to acidemia from controls • Use of statistical tests and metrics (e.g., ROC curves and AUROC values)	• The highest AUROC values were found when the UA pH threshold was set equal to 7.05, causing the highest imbalance, and used in combination with different options for the LF range • High AUROC values were also found with a UA pH threshold equal to 7.1 always in combination with the LF range (e.g., an AUROC value equal to 0.73 was found using a 0.04–0.15 Hz LF range)	• Only a limited number of CTG tracings leading to acidemia was included when lower thresholds for acidemia were considered
Georgieva et al. (2014) [27]	• 7568 total fetuses (mean GA = 39.8 weeks) including the following: - 319 with acidemia (UA pH ≤ 7.05) - 7249 controls • Use of CTG tracings from the active second stage of delivery	• Investigation of the predictive power for acidemia associated with deceleration capacity (DC) and acceleration capacity (AC), fHRV features derived from the PRSA • Use of statistical tests and metrics (e.g., ROC curves and AUROC values)	• AC and DC performed similarly and showed a high correlation • AUROC values found using DC were shown to be superior to the one obtained with STV (mean AUROC value = 0.67 vs. 0.61)	• Only a comparison between individual fHRV features, resulting in relatively low mean AUROC values, and no multivariate model was considered
Rivolta et al. (2020) [29]	• 465 total CTG tracings (GA ≥ 37.0 weeks) from the CTU-UHB dataset including the following: - 24 with acidemia (UA pH ≤ 7.05) - 441 controls • Use of CTG tracings from the last 1 h before the onset of delivery stage II	• Introduction of the deceleration reserve (DR), a fHRV feature derived from the PRSA • Investigation of their predictive power for acidemia associated to DR, DC, and AC • Use of statistical tests and metrics (e.g., ROC curves and AUROC values)	DR, introduced to estimate possible asymmetric increasing or decreasing fHR trends, returned a higher mean AUROC, equal to 0.65, compared to AC and DC	• Only a limited number of CTG tracings leading to acidemia was included • Only a comparison between individual fHRV features, resulting in relatively low mean AUROC values, and no multivariate model was considered

Additional investigation of the predictive power associated with time-domain fHRV features was performed by Gatellier et al., who investigated a total of 439 CTG tracings from the CTU-UHB dataset, including 43 resulting in acidemia (UA pH < 7.10) [102]. The authors limited their analysis to two 5 min periods from the last 90 min before delivery. These included a stable fHR period, characterized by an absence of accelerations and decelerations as well as by a low fHRV, and the period characterized by maximum Fetal Stress Index value (FSI_max_). FSI was computed by filtering the fHR to only maintain oscillations above 0.15 Hz, by plotting a lower and upper envelope function constructed by considering local minima and maxima, and by consequently identifying the lowest enclosed area among a set of four areas, each found by considering 16 s windows and no overlap. Different time-domain fHRV features were computed in this study, including STV and FSI. Since the latter only allows considering high-frequency oscillations, it has been speculated that, much like the STV, it allows to estimate the contribution of the PNS to the fHRV [102,105]. Higher values for different fHRV features, such as STV and FSI, were identified in CTG tracings leading to acidemia in both 5 min periods, despite significance only being found when considering the first fHRV feature and the period characterized by FSI_max_. This result reflects an increase in parasympathetic modulation for selected short periods for fetuses developing acidemia that are close to term.

The use of the FSI was portrayed in other studies, including one from Butruille et al. [105]. They investigated a total of 299 CTG tracings, out of which 12 were derived from fetuses at risk of acidemia (defined based on UA pH thresholds for different GA [106]). The FSI was continuously computed over the last 30 min before delivery using a time shift of 1 s for moving windows, allowing the computation of several statistics for this fHRV feature, such as FSI_min_ and FSI_mean_. Significantly lower values for both were found in CTG tracings leading to acidemia, a contrasting result to the one found by Gatellier et al. [102], possibly due to the choice of investigating different periods.

#### 3.3.2. Frequency-Domain fHRV Features

Frequency-domain fHRV features are computed using the power spectral density (PSD) with either non-parametric or parametric methods, such as the Fast Fourier Transform or autoregressive models, respectively [26,53,71,105,107]. These require the definition of spectral bands, indicating the frequency ranges that are associated with the modulation of the different branches of the fetal ANS as well as with fetal breathing and movements. However, no consensus has been reached yet on how to separate the spectral bands and how many to use.

One of the first important insights regarding this matter was provided in a systematic review by Van Laar et al., who investigated strategies to perform frequency-domain analysis of the fHRV in the context of fetal surveillance [26]. The authors collected indications and results from four intrapartum studies hinting at a correlation between frequency-domain fHRV features and acidemia based on different definitions (e.g., UA pH < 7.05 or UA pH < 7.2) and derived CTG tracings from either Doppler ultrasound or a scalp electrode. In addition to this, a variety of options to separate the spectral bands were adopted in the included studies (e.g., the low-frequency (LF) range was located in the range 0.08–0.15 Hz in two studies). All the included studies used absolute values to describe the PSD. Most studies pointed out that a general decrease in the PSD in the LF range can be noticed in fetuses developing acidemia, indicating a decrease in the modulation of both branches of the ANS [26,108]. However, the included study by Salamalekis et al. reported an increase in the PSD in the LF range for fetuses developing acidemia [109]. Since they used a higher threshold to discriminate acidemic fetuses from controls (i.e., UA pH < 7.2), this result can indicate that the severity of acidemia may influence this fHRV feature, as stated by Van Laar et al. [26]. Another included study, performed by Siira et al., investigated different fHRV features including the PSD in the LF range and the ratio between the power spectral density computed in the low- and high-frequency range (LF/HF ratio) [108]. These authors identified a peculiar pattern for these fHRV features only in fetuses developing acidemia. An initial increase in the values of both fHRV features was followed by a decrease in the PSD within the LF range closer to birth. The increased stress experienced by fetuses following the start of uterine contractions could be responsible for the initial increase in PSD in the LF range caused by sympathetic modulation, which is then followed by cardiovascular decompensation [26,108]. The review by Van Laar et al. also indicated that the reliability of the PSD in the high-frequency (HF) range computed by considering CTG tracings derived from Doppler ultrasound could be affected by the low sampling frequency and potentially violate the Nyquist–Shannon sampling theorem. This limitation implies that the PSD in the HF range cannot be properly estimated if averaging of consecutive beat-to-beat intervals takes place. As a consequence, the authors suggested careful consideration when selecting the upper limit for the usable frequency range.

Van Laar et al. also investigated these findings in a follow-up study, aiming to predict acidemia using frequency-domain fHRV features while considering the last 3 h before delivery in 10 cases (defined as UA pH < 7.05) and 10 controls with similar characteristics [71]. The PSD in the LF and HF ranges (LF = 0.04–0.15 Hz, HF = 0.4–1.5 Hz) was computed using 64 s moving windows, time shifts of 0.25 s, and both absolute and relative values. The latter values, in particular, were obtained by normalizing with the total PSD (TP = 0.04–1.5 Hz). Both fHRV features computed by using relative values were found to be statistically significant when comparing the two included groups of fetuses during the last 30 min before delivery. Higher values for the relative PSD in the LF range were found in fetuses developing acidemia, while higher values for the relative PSD in the HF range were found in controls. This result, which was later confirmed in a follow-up study [110], constituted the first evidence that the minutes preceding the onset of acidemia are characterized by an overall decrease in the modulation of the ANS (i.e., visible using absolute values for the PSD), with a predominance of the SNS (i.e., visible using relative values for the PSD).

In a recent systematic review focused on the frequency-domain analysis of the fHRV in the intrapartum period and acidemia, Castro et al. evaluated the performance of all the spectral bands derived from previous studies [28]. These included, for instance, the very low-frequency (VLF) range and the mid- or movement frequency (MF) range, together with the LF and HF ranges. The evaluation was conducted on a subset of the CTU-UHB dataset, comprising a total of 246 CTG tracings, while portraying four different UA pH thresholds, ranging from 7.05 to 7.20, to discriminate between acidemia fetuses and controls. The authors identified a significant increase in the PSD in the VLF range and a significant decrease in the PSD in all the other ranges in fetuses developing acidemia compared to controls using different UA pH thresholds. High AUROC values were, for instance, found by using different options for the LF range and a UA pH threshold equal to 7.1. This returned an AUROC value equal to 0.73 using a 0.04–0.15 Hz LF range, a choice which had frequently been used in previous studies [71,108,111]. These results indicated that fHRV features derived using PSD have the potential to meaningfully contribute to the prediction of acidemia when used as an adjunctive method to CTG.

#### 3.3.3. Non-Linear fHRV Features

Non-linear fHRV features are commonly used to measure irregularity within the fHR and can be useful to quantify changes provided by different regulatory mechanisms, in a similar way to what is commonly performed considering newborns or adults [28,112]. fHRV features derived from the phase-rectified signal averaging (PRSA) fall into this group [113,114]. PRSA is a method that allows for the detection and quantification of quasi-periodic oscillations within the fHR. It consists of two main steps: (1) the identification of anchor points corresponding to either increase or decrease events in the original time series, performed by comparing sums of T values preceding and following a candidate anchor point, and (2) signal averaging, where windows of length 2 L are defined around each anchor point, aligned, and consequently averaged. Different fHRV features can then be computed depending on the original choice to consider increase or decrease events. These include the acceleration capacity (AC) and deceleration capacity (DC), which quantify the central spike of the PRSA curve based on the number of points preceding and following the anchor point, as indicated by a preselected value for the scale parameter s. Although it has recently been shown that the AC and DC cannot be directly related to the sympathetic and parasympathetic modulation [27,29], they can be related to the STV since they estimate rapid changes in the modulation of the fHR.

Georgieva et al., in particular, investigated which of these three fHRV features performed better in predicting acidemia in a total of 7568 fetuses, of which 319 indeed developed this condition (e.g., defined as UA pH ≤ 7.05) [27]. AC and DC were computed with different combinations of the previously mentioned parameters T and L. As a result of this study, AC and DC performed similarly and showed a high correlation. Irrespective of the choice that was performed for the two parameters, AUROC values found using DC were shown to be slightly superior to the one obtained with STV (mean AUROC values equal to 0.67 vs. 0.61).

The use of PRSA applied to the fHR to predict acidemia was also found to be successful in a recent study performed by Rivolta et al. [29]. These authors introduced a new fHRV feature, the deceleration reserve (DR), defined as the difference between DC and AC, aiming at identifying possible asymmetric increasing or decreasing fHR trends. DR was used to predict acidemia in a subset of the CTU-UHB dataset, including a total of 465 CTG tracings out of which 24 were characterized by this condition (i.e., defined as UA pH ≤ 7.05). DR was compared with AC and DC and returned the highest mean AUROC, equal to 0.65, providing further indication that fetuses developing acidemia are characterized by different autonomic regulation.

#### 3.3.4. Summary

In summary, the main advantage of using different fHRV features compared to the different guidelines for CTG interpretation and fHR baseline, deceleration-, and acceleration-derived features is the ability to investigate the modulation of the two branches of the ANS in fetuses developing acidemia compared to controls [26,28,71,105,108].

**Figure 2:**
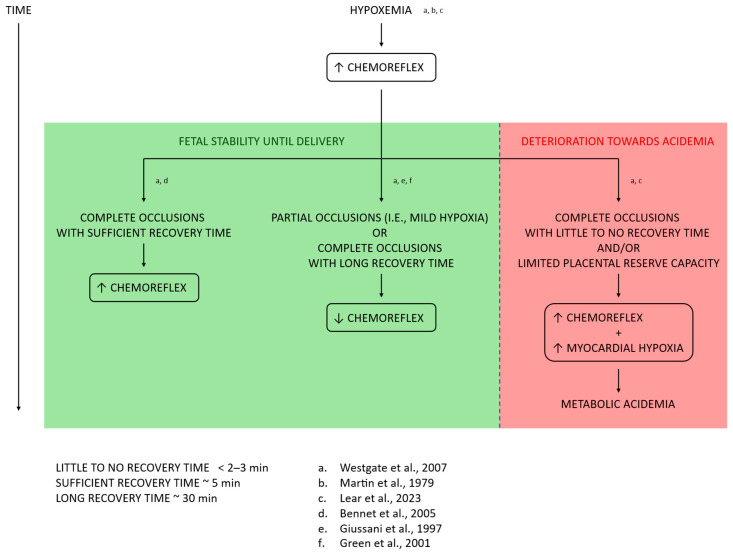
Conceptual model of the key changes occurring to the modulation of the autonomic nervous system (ANS) of fetuses developing acidemia with respect to controls during the intrapartum period. These results were obtained from various invasive studies of fetal sheep by the authors indicated within this Figure [104,115,117,118,119,120].

Results presented in this section can therefore be related to findings from several invasive animal studies, including those on fetal sheep, where umbilical cord occlusions were induced to observe the effects from the onset of hypoxemia to the onset of metabolic acidemia [115,116,117,118,119,120]. Although some of these findings remain debated, they have shed some light on ANS mechanisms that are more likely to occur under certain conditions in the intrapartum period. A literature review by Westgate et al., in particular, summarized many of these findings [104], which are also reported in Figure 2 and further described in this section.

As it was described for the first time by Martin et al. in 1979 and confirmed in different following studies, the onset of a hypoxic condition and consequent hypoxemia activate the chemoreflex [115,116,117]. This stimulates the modulation of the PNS and SNS [115], which are influenced in this order with only a short delay between them [121]. Hypoxemia can then progress in different ways depending on several factors, including but not limited to the frequency of uterine contractions, the recovery period between occlusions, and the placental reserve capacity. The latter, in particular, determines how quickly the placenta can restore the oxygenation levels. However, three cases have been reported and discussed in different studies [116,117,118,119,120], leading to a general consensus among healthcare professionals regarding the underlying physiological mechanisms. In the first case, described by Bennet et al. [118], complete occlusions with sufficient recovery time (i.e., 1 min occlusions, repeated every 5 min) of the umbilical cord result in the chemoreflex remaining dominant. In the second case, partial occlusions of the umbilical cord (i.e., leading to a 50% reduction in cord blood flow) [119] or long recovery time between even longer complete occlusions (i.e., 90 s occlusions, repeated every 30 min) [120] result in an attenuation of the modulation of the chemoreceptor. In both cases, no significant changes in the recorded arterial pH values were identified compared to the start of each experiment. The third case can instead be encountered in the presence of frequent complete occlusions with little to no recovery time (i.e., 1 min occlusions, repeated every 2.5 min), which progressively leads to the development of metabolic acidemia and to a fall in arterial pH. As recently found by Lear et al. [117], during this occurrence, both the chemoreflex and myocardial hypoxia influence the ANS. This leads to increased modulation of SNS activity and the release of various humoral factors, such as adrenal catecholamines, to sustain metabolic needs.

The predominant modulation of the SNS with respect to the PNS in acidemic fetuses in the hours leading to the delivery, shown by Van Laar et al. with an increase in the relative PSD in the LF range [71,110], is therefore in line with the results derived from invasive animal studies reaching the onset of acidemia [117]. Consistent with these findings, Butruille et al. also observed a decreased modulation of the PNS in these fetuses, evidenced by a reduction in FSI_min_ and FSI_mean_ [105]. The decrease in the absolute PSD in the LF range in acidemic fetuses appeared as the most frequent result among the included studies [26,108]. This result indicates an overall attenuation of the modulation of the ANS and might result from decompensation and the inability of the fetus to sustain the physiological systems due to a lack of metabolic reserves. Further discussion of results obtained using various fHRV features, combined with comparisons to findings from invasive animal studies, could help to better elucidate the physiological mechanisms underlying the onset of acidemia.

Additional considerations that can be derived from this section include the fact that choices for the best spectral bands to be used for the frequency-domain fHRV features to discriminate acidemia fetuses from controls are also still debated. In particular, the ranges 0.03–0.07 Hz and 0.04–0.15 Hz appear to be the most promising choices for the PSD computed in the LF range, depending on the UA pH threshold that is used [28]. fHRV features derived from the PRSA, such as AC and DC, which provide an estimate of rapid oscillations in the fHR, and DR, which quantifies signal asymmetries, provided promising results for the prediction of acidemia and outperformed STV [27,29].

### 3.4. ST Events

The use of the scalp electrode represents the gold standard in clinical practice for the computation of ST events, a term used to describe changes occurring to the ST segments of the fECG [30,31,32,33,34,35,36]. ST events assess changes in the T/QRS amplitude ratio and the presence of a biphasic ST waveform, both of which serve as indicators of an impending risk of acidemia when they coincide with non-reassuring CTG tracings [30,31,36]. T/QRS amplitude ratio enables the detection of an increase in T wave height, which occurs when the oxygen supply is insufficient to sustain aerobic metabolic activity, resulting in energy production through anaerobic metabolism [30,31]. ST depression accompanied by a biphasic ST waveform, characterized by a negative slope before the onset of the T wave, can also occur in the presence of hypoxia. This reflects an imbalance between the mechanical performance of the endocardium and epicardium (i.e., the inner and outer layers of the heart muscle, respectively), with the first being characterized by a slower repolarization, due to decreased blood flow reaching the endocardium and increased mechanical strain [30,31]. Since the early 2000s, different commercial devices such as different iterations of the STAN and SisPorto systems [55,68] relied on ST events to try to improve the prediction of acidemia in clinical practice. Different studies that assessed their performance are included in this section and summarized in Table 5.

An important study that made use of the SisPorto and ST events was performed by Costa et al., who aimed at predicting acidemia while involving a total of 148 fetuses, out of which 7 were diagnosed with this condition (defined as UA pH ≤ 7.05) [55]. Results of this study, which included the last 1 h of CTG tracings before delivery, showed that ST events provided additional complimentary information to other fHR features and that they increased the sensitivity almost without affecting the specificity (SE = 1.00 vs. 0.57, SP = 0.94 vs. 0.97). Nonetheless, the low precision obtained even when including ST events (PRE = 0.47 vs. 0.50) represents an important limitation of this approach, which introduces several false alarms [35,40].

Several studies were also performed to verify whether ST events provide additional value compared to fHR features alone when used to predict the occurrence of different fetal conditions as well as unnecessary operative deliveries [32,33,122]. The introduction of the scalp electrode and ST events in clinical practice were also assessed by Landman et al. [56], who performed a follow-up study to a large randomized controlled trial (RCT) conducted in the Netherlands just a few years before [32]. These authors saw an 84% reduction in the rate of acidemia, when evaluating the effects of the introduction of the scalp electrode in clinical practice over a period of 14 years and a total of 19,664 fetuses [56]. The authors identified two possible reasons for the observed improvement in the rate of acidemia: (1) the introduction of the scalp electrode and ST events in clinical practice, and (2) the intensified training of the hospital personnel.

Different reviews also debated whether the use of internal fetal monitoring and the derived ST events can aid clinicians in reducing the incidence of acidemia. A recent review written by Cagninelli et al. investigated all RCTs and meta-analyses of RCTs published until 2020 presenting the effects associated with the use of ST events in clinical practice [59]. The key result that can be derived from this review is that conflicting results were presented regarding potential benefits of using ST events to reduce the occurrence of acidemia. According to the authors, this result could be due to the use of different definitions for this condition. A more recent systematic review from Tsiligkeridou et al. confirmed the presence of conflicting results derived from previous RCTs [60]. Therefore, these studies did not recognize the superiority of combining ST events with Doppler ultrasound over using Doppler ultrasound alone in aiding clinicians to prevent the onset of acidemia.

A possible explanation regarding these conflicting results and the high rate of false alarms was presented by Hulsenboom et al., who pointed out that the scalp electrode is unfortunately only capable of representing the electrical activity of the heart in one dimension, as it only provides a single lead fECG [36,123,124]. Because of this reason, the amplitude of the different fECG waves reflects the projection of electrical potential in the direction of this single lead. Since the traditional computation of the T/QRS does not take into account the fact that the propagation direction in the depolarization phase (QRS complex) is not parallel (or antiparallel) to that in the repolarization phase (T top), this can lead to an increase in false positive as well as false negative ST events [36]. To solve this limitation, Hulsenboom et al. proposed the use of relative ST events [35,36,124]. These are defined by computing the ratio between T/QRS amplitude ratios and their respective baseline, instead of a difference between the two, as a way to compensate for inter-patient differences in the electrical heart axis and to reduce detection errors. In addition to this, the authors identified a 70% rise above the baseline as an optimal threshold for the definition of relative ST events. In the most recent study performed by the same authors, which involved 10 fetuses developing acidemia (defined as UA pH < 7.05) and 10 matched controls, relative ST events were compared with absolute ST events, defined using the traditional definition, [36,124]. Relative ST events returned higher sensitivity and specificity in predicting fetuses developing acidemia compared to absolute ST event (SE = 0.90 vs. 0.70, SP = 1.00 vs. 0.70) despite statistical analyses not showing significant differences between the two strategies, possibly due to the low sample size.

Very recently, despite not focusing on any specific feature from the fetal scalp electrode, Tarvonen et al. examined the relationship between CTG tracings recorded during labor and different fetal and neonatal complications [57]. These CTG tracings were obtained using three different strategies: a Doppler ultrasound transducer, a combination of Doppler ultrasound transducer and a maternal heart rate (mHR) transducer, and a scalp electrode. This study analyzed a large Finnish cohort of 213,798 fetuses, with a fairly balanced distribution among the three groups. The authors also separated the included fetuses considering different parameters, such as UA pH, base excess, and 5 min Apgar score, as well as others, and indicated that a total of 627 fetuses presented a UA pH < 7.0. The findings revealed that fetuses monitored with Doppler ultrasound transducer had a 2.2-fold increased risk of acidemia (when this was defined as UA pH < 7.0) compared to those monitored with the other two strategies. These differences remained significant even after adjusting for maternal, delivery, and fetal risk factors, with only marginal attenuation of the associations. Moreover, fetuses monitored using a combination of Doppler ultrasound transducer and a mHR transducer had a 1.4-fold increased risk of acidemia defined by composite criteria (including at least two parameters such as UA pH, base excess, and 5 min Apgar score, as well as others) compared to those monitored using a scalp electrode. Given the large sample size, this study suggests that fetal monitoring via the scalp electrode could still hold benefits. A follow-up study on the same cohort including features such as ST events would therefore help clarify their clinical value.

In the past years, healthcare professionals expressed some doubts regarding benefits provided using internal monitoring strategies from which ST events are derived. These doubts stem from the invasive nature of the scalp electrode and its restricted applicability, which is limited to third-trimester fetuses (GA ≥ 32 weeks) during the labor period, in case of sufficient cervical dilation, and after membrane rupture [10,30,31,38,40]. In addition to this, different reviews and RCTs have long debated whether the use of ST events can lead to a reduction in the incidence of acidemia, and conflicting results have been reported [56,59,60]. It has been indicated that promising results achieved using ST events might also be influenced by additional factors, such as intensified training of hospital personnel [56]. Future studies with large populations are needed to verify whether relative ST events [36,124] can significantly outperform absolute ST events. Such findings could offer a compelling reason for more regular use of the scalp electrode in clinical practice during the intrapartum period.

### 3.5. Machine Learning and fHR Features

In recent years, with the growing popularity of machine learning in the medical context, different prediction models using fHR features were developed to predict acidemia [14]. This section includes selected studies organized following the presentation order of fHR features in previous sections (i.e., studies using machine learning and fHR baseline, deceleration-, and acceleration-derived features are presented first, followed by those using fHRV features) and increasingly more complex machine learning as well as deep learning algorithms. A summary of the results of these studies is also included in Table 6.

Cahill et al. used both backward stepwise logistic regression and log-binomial regression models to demonstrate that the total deceleration area, combined with various labor and delivery conditions, can achieve higher performance compared to models using different NICHD categories and other fHR features (e.g., fHR baseline) [24,44]. The most recent promising model was computed by considering 149 fetuses developing acidemia and 8431 controls and returned the highest AUROC value and values for most metrics (AUROC = 0.76, SE = 73.5%, SP = 67.2%, PRE = 4.0%, negative predictive value (NPV) = 99.3%) [44]. In addition to this, a small boost in performance was obtained by including tachycardia in the previous prediction model (AUROC = 0.77, SE = 66.0%, SP = 76.2%, PRE = 5.0%, NPV = 99.2%). However, the very low precision values obtained in this study limit the use of such models in clinical practice since they could expedite delivery in a very high number of controls and consequently lead to an increase in the number of unnecessary cesarean sections, with associated risks and costs.

Ekengård et al. used logistic regression together with fHR acceleration-derived features to predict acidemia in an extensive population that consisted of 364 cases (defined as UA or UV pH < 7.05 at vaginal birth or pH < 7.10 at birth after first-stage cesarean delivery) and 731 controls [46]. These authors showed that the presence of two or more sporadic accelerations was a significant predictor for the absence of acidemia, especially when considering the first stage of labor, which lasts up to full cervical dilation. On the other hand, only a weak association between the presence of isolated periodic accelerations and the onset of acidemia was found by considering the second stage of labor. However, this study is characterized by important limitations. These include the fact that the healthcare professional annotating the accelerations was not blinded to the fetus group, as well as the use of a fixed threshold to discriminate between the counts of sporadic and periodic accelerations.

Several authors employed a variety of machine learning and deep learning algorithms on the CTU-UHB dataset, a choice which allowed a fair comparison of the obtained results [45,125,126,127]. One shared limitation of the mentioned studies relates to the lack of validation of the results on an external dataset. Moreover, the use of an UA pH < 7.15 as a cut-off to define acidemia included in some of these studies has received little support from the literature [13].

Xiao et al. included not only the CTU-UHB dataset but also a second private dataset, composed of a total of 784 CTG tracings, which was only used for external validation [15]. This study, which also used the previously mentioned UA pH < 7.15 to identify fetuses developing acidemia, introduced multiple prediction models. These made use of time-domain and non-linear fHRV features (e.g., support vector machines (SVM) and logistic regression used fHRV features such as STV and sample entropy), which were directly encoded from raw CTG tracings (e.g., using a multiscale CNN based on bidirectional long short-term memory network (BiLSTM)) or a combination of both (e.g., a deep feature fusion network (DFFN)). In addition to this, stratified 10-fold cross-validation was also included. Within their study, the authors compared their results with the ones obtained by reproducing the prediction models from previous authors who worked with the CTU-UHB. This was performed by using only CTG tracings derived from the 20 min preceding the delivery. Since the choice of the considered period differed compared to the one considered in the original studies from which these prediction models were derived, this led to different metrics values. Using these extensive experiments, Xiao et al. verified that the performance of their DFFN outperformed the other strategies, both on the CTU-UHB dataset (SE = 0.62, SP = 0.74, quality index (QI) = 0.67) and on the second private dataset (SE = 0.44, SP = 0.66, QI = 0.54). Nonetheless, the lower values achieved with the second private dataset reflect the difficulties that are commonly found when transferring the learned knowledge to a different dataset and indicate that additional efforts will be needed to develop a more generalized prediction model able to work in different clinical settings.

**Table 6:** Overview of studies using prediction models using fetal heart rate (fHR) features to predict acidemia.

Study	Population and CTG Tracings	Methods	Key Results	Distinct Limitations
Cahill et al. (2018) [44]	• 8580 total fetuses including the following: - 149 with acidemia (mean GA = 39.0 weeks, UA pH < 7.10) - 8431 controls (mean GA = 39.0 weeks) • Use of CTG tracings from the last 120 min before delivery	• Investigation of the association between CTG tracings and fHR features, including total deceleration area, alone or in combination, with acidemia and neonatal morbidity • Use of statistical tests and prediction model (e.g., log-binomial regression)	• The use of total deceleration area used in combination with labor and delivery conditions returned the highest AUROC value and values for most metrics (AUROC = 0.76, SE = 73.5%, SP = 67.2%, PRE = 4.0%, NPV = 99.3%) • Tachycardia, best fHR feature derived from the NICHD 3-tier clinical guideline, provided little additional value to the prediction model (AUROC = 0.77, SE = 66.0%, SP = 76.2%, PRE = 5.0%, NPV = 99.2%)	• The included population was characterized by a severe imbalance • A very low precision value was returned by the prediction model
Ekengård et al. (2023) [46]	1095 total fetuses (GA ≥ 34.0 weeks) including the following: - 364 with acidemia (UA or UV pH < 7.05 at vaginal birth or pH < 7.10 at birth after first-stage cesarean delivery) - 731 controls • Use of CTG tracings from the last 30–60 min before delivery for acidemic fetuses, from the last 30–60 min before the first or second stage of labor (to provide a match) for controls	• Investigation of possible associations between sporadic and periodic fHR accelerations and acidemia • Use of a prediction model (i.e., logistic regression)	• The presence of ≥2 sporadic accelerations significantly predicted the absence of acidemia in the first two stages of labor • Periodic accelerations only showed a weak association with acidemia during the second stage of labor	• The healthcare professional that annotated accelerations was not blinded to the fetus group • Fixed threshold used to discriminate between the counts of sporadic and periodic accelerations
Xiao et al. (2022) [15]	• 552 total CTG tracings (mean GA = 40.0 weeks) from the CTU-UHB dataset including the following: - 113 with acidemia (UA pH < 7.15) - 439 controls • 784 total CTG tracings (mean GA = 39.0 weeks) from a second private dataset • Use of CTG tracings from the last 20 min before delivery	• Prediction of acidemia using time-domain and non-linear fHRV features as well as features directly derived from CTG tracings using convolutional kernels • Use of prediction models and metrics (e.g., deep feature fusion network (DFFN)) • Comparison with the results achieved with previous prediction models	• DFFN outperformed other prediction models considered by the authors as well as prediction models from previous authors who worked with the CTU-UHB • Values for different metrics were found to be superior both while considering the CTU-UHB dataset (SE = 0.62, SP = 0.74, quality index (QI) = 0.67) and the second private dataset (SE = 0.44, SP = 0.66, QI = 0.54)	• The lower values for the metrics achieved with the second private dataset indicate a poor transfer of the learned knowledge • Different choices performed by the authors reduced values for the metrics compared to the ones found in the original studies
Francis et al. (2024) [14]	• 9923 total fetuses from 36 studies • Use of different definitions for acidemia (e.g., UA pH ≤ 7.05) • Use of intrapartum CTG tracings	• Scoping review investigating how machine learning has been used to analyze CTG tracings and predict acidemia • Investigation of the used datasets, machine learning algorithms, and fHR features • Systematic search and screening performed based on eligibility criteria to ensure an unbiased selection of studies	• The use of different definitions for acidemia, datasets, and fHR features prevented a direct comparison of the results achieved in different studies	• Inclusion only of studies investigating the intrapartum period, published in English, after the year 2000, and in journals
Ben M’Barek et al. (2025) [128]	27,662 total fetuses (GA ≥ 37.0 weeks) from five different datasets, Including the following: • 464 with severe acidemia (UA pH ≤ 7.05) • 3457 with moderate acidemia (7.05 < UA pH ≤ 7.05)	• Prediction of acidemia using a CNN, pretrained to estimate relevant FHR features, and then trained on four datasets and tested on the other (leave-one-group-out cross-validation)	• The prediction model returned moderate performance scores with some variations among datasets and level of acidemia. • The best performance (AUROC = 0.81) was achieved on the “Robert Debrè” private dataset, while predicting severe acidemia, • The worst performance (AUROC = 0.70) was achieved on the CTU-UHB dataset, while predicting moderate and severe acidemia	• Lower performances compared to other studies

A recent scoping review provided an overview of how machine learning has been used to analyze CTG tracings and predict the onset of acidemia [14]. A total of 36 studies were identified by Francis et al. The most frequently used algorithms included SVM, CNN, k-nearest neighbors, decision trees, and random forests. Of these, 22 studies used the CTU-UHB dataset, and only 4 performed a validation of the prediction results using an external dataset. In addition to this, different definitions were used to discriminate acidemic fetuses from controls, and fHR features varied significantly among studies despite all including the FIGO 3-tier clinical guideline. All these differences prevented a direct comparison of the results achieved in different studies. A new public dataset consisting of CTG tracings and annotations for perinatal outcomes is considered by the authors of this scoping review as an important necessity for future studies, since it can provide a benchmark to compare prediction models and further indication regarding the generalizability of the prediction results.

More recently, M’Barek et al. trained and validated a CNN using five different datasets, one of which was the CTU-UHB dataset, and included a total of 27,662 fetuses [128]. Among these, 464 developed severe acidemia (defined as UA pH ≤ 7.05) and 3457 moderate acidemia (defined as 7.05 < UA pH ≤ 7.20). Their prediction model was trained and validated using leave-one-group-out cross-validation, allowing for a fair external validation, and returned AUROC values ranging from 0.70 to 0.83 when predicting moderate and severe acidemia and from 0.74 to 0.81 when predicting severe acidemia alone.

To summarize, in recent years there has been an increasing trend in using machine learning as well as deep learning to predict the onset of fetal acidemia. Different prediction models were created by using a wide range of fHR features, spanning from fHR baseline, deceleration-, and acceleration-derived features to fHRV features [14,44,46]. In addition to this, the use of CNN allowed the computation of new sets of features and led to high prediction results, evaluated while considering different metrics [14,45,129]. Ultimately, the combination of both approaches (i.e., the use of fHR baseline, deceleration-, and acceleration-derived features, fHRV features, as well as features derived using a CNN) seems to be a promising direction to further improve the prediction results [14]. The use of more extensive and balanced datasets can provide further indications about the validity of these approaches.

## 4. Discussion

The main objective of fetal monitoring during the intrapartum period is to identify fetuses that are not adequately oxygenated and are therefore at risk of developing acidemia [3,10]. Through the years, several studies involving clinical guidelines for CTG interpretation, fHR features, and ST events were employed to achieve this objective. A selection of these studies was included in this literature review to provide an overview of the most common and promising approaches for acidemia prediction while considering important limitations.

Clinical guidelines for CTG interpretation define the risk of fetal complications based on different fHR baseline, deceleration-, and acceleration-derived features, as well as fHRV features [10,16,20,21]. Due to their high interpretability, they have been widely adopted into clinical practice and integrated into various commercial devices aiming to perform an automated investigation of CTG tracings [19,61]. However, multiple studies have questioned the utility of clinical guidelines for CTG interpretation in predicting the onset of fetal complications, as they frequently return intermediate risk annotations, leaving the final decision regarding fetal management to the clinicians [74,76,77]. Additionally, intermediate risk annotations derived from different clinical guidelines for CTG interpretation returned conflicting results for the sensitivity and specificity metrics across different studies [74,80], making it difficult to identify which can work best in clinical practice.

The use of fHR baseline and deceleration-derived features indicated that even fHR features characterized by a simple computation, such as total deceleration area and short saltatory patterns, can contribute to the prediction of acidemia since consistent results were found in different studies [23,24,77,84,85,86]. On the other hand, the use of different fHRV features, derived from both the time- and frequency-domain not only provided an effective means of identifying acidemic fetuses but also a way to investigate the functioning of the two branches of the ANS [26,28,71,102,105,108,109,110]. Changes in various fHRV features, such as the FSI and PSD in the LF range [26,71,105,108,110], can also be related to findings from invasive animal studies [115,116,117,118,119,120] to assess the severity of umbilical cord occlusion and determine whether the physiological systems of the fetus are still able to sustain the metabolic demands after the onset of acidemia. Moreover, only a limited number of studies quantified changes occurring to these fHR features over time in the hours leading up to delivery [24,84,105,108].

The use of ST events in clinical practice is still debated by different authors to this day. This is not only due to considerations related to the invasiveness of the necessary scalp electrode and the associated risks [36,55,56] but also to the lack of consistent results from reviews and RCTs supporting the introduction of the scalp electrode as a way to improve prediction of acidemia [56,59,60].

The use of machine learning and fHR features is one of the most widely used strategies to predict acidemia in recent years [14,15,45,129]. This is because prediction models developed following this strategy can consider the inclusion of multiple fHR baseline and deceleration-derived features, fHRV features, as well as features implicitly computed by deep neural networks to enhance the predictive power for acidemia [14,15,45,129]. Machine learning also allows to rank the relevance of each individual fHR feature. Since different studies used various sets of fHR features, the ones that were ranked higher in terms of importance also differed (e.g., total deceleration area, presence of ≥2 sporadic accelerations) [44,46,77]. In addition to this, studies that included a CNN returned high values for several metrics [45,129], a result that shows how fHR features that are exclusively computed using this type of neural network can be promising to improve the prediction of acidemia. It is, however, important to mention that most studies using prediction models based on machine learning report standard performance metrics but do not perform temporally continuous evaluations to assess how acidemia predictions evolve over time. Additionally, several studies made use of the same public dataset (i.e., the CTU-UHB dataset), a solution that enabled a fair comparison of the results achieved with different prediction models and allowed the achievement of high values for various metrics [14,15,45,129] but at the cost of limiting the generalizability of these findings to broader clinical populations.

## 5. Future Directions

Advancing the prediction of fetal acidemia requires a holistic approach that integrates physiological understanding, improved monitoring technologies, and refined clinical decision-making frameworks.

Future research should explore the inclusion of additional fHR features within clinical guidelines for CTG interpretation to reduce the current ambiguity in clinical decision-making [74,76,77]. Establishing a consensus on which fHR features provide the most robust predictive value could inform updates to international standards and automated CTG interpretation systems. Beyond the refinement of clinical guidelines for CTG interpretation, bridging the gap between statistical observations and physiological mechanisms remains essential. While fHRV features show promise, correlating these non-invasive metrics with findings from invasive animal studies [115,116,117,118,119,120] is relevant to validate their relationship with umbilical cord occlusion severity and metabolic compensation. Future studies should quantify changes in fHRV features over time, specifically in the hours leading to delivery, to capture the progression of deterioration.

The invasiveness of scalp electrodes remains a barrier to widespread adoption to integrating ST event monitoring into standard practice [36,55,56]. Further studies making use of relative ST events, introduced as a way to compensate for limitations introduced using a single lead fECG [35,36,124], could better justify their use. Future improvements in transabdominal monitoring strategies based on electrophysiology could further allow for the computation of ST events without the need for an invasive electrode, as this has already been performed [130], and such an approach could potentially be extended to the antepartum period.

Concurrently, studies employing machine learning and fHR features should move beyond standard metrics derived from static datasets. Performing evaluations over continuous periods could therefore provide additional information to complement those derived from invasive animal studies and mathematical simulations and possibly further contribute to obtaining new insights about fetal responses to the onset of acidemia. Future studies should also dedicate more attention to validating their results using external datasets since, until now, those few studies that performed external validation have shown lower performance [14,15]. Proper external validation is key to verify the generalizability of a prediction model, defined as the ability to transfer knowledge derived from one dataset to a different one, while also enabling the identification of factors that may limit or require adaptation of such generalizability, provided that the characteristics of the populations and clinical settings (e.g., fetal characteristics, choices adopted during labor/delivery as well as interventions, information regarding drug administration), as well as the definition of the condition to investigate, are comparable or their differences can be directly examined. This information should therefore be well documented when collecting CTG tracings in new datasets. Future efforts should also focus on expanding the availability of public datasets and further validating predictive models across diverse populations. This will ensure more robust and generalizable findings, ultimately enhancing perinatal outcomes in clinical practice. Additionally, there is an urgent need to address non-unique definitions for acidemia to better compare results from different studies.

## Figures and Tables

**Table 5:** Overview of studies using ST events to predict acidemia.

Study	Population and CTG Tracings	Methods	Key Results	Distinct Limitations
Costa et al. (2009) [55]	• 148 total fetuses (median GA = 39.0 weeks) including the following: - 7 with acidemia (UA pH ≤ 7.05) - 141 controls • Use of CTG tracings from the last 1 h before delivery	• Use of the SisPorto to predict acidemia • Comparison of the predictive power associated with fHR features and with fHR features plus ST events • Use of metrics (e.g., sensitivity and specificity)	• ST events provided an increase in sensitivity, a similar specificity, but did not improve low precision (SE = 1.00 vs. 0.57, SP = 0.94 vs. 0.97, PRE = 0.47 vs. 0.50)	• A very low precision value was returned even when ST events were considered • Only a limited number of fetuses developing acidemia was included
Landman et al. (2019) [56]	• 19,664 total fetuses (GA ≥ 36.0 weeks) including the following: - 230 with acidemia (UA pH < 7.05 and BD > 12 mmol/L) (estimated value based on the reported percentages) - 19,434 controls • Use of CTG tracings after rupture of membranes	• Investigation of the effects of the introduction of the scalp electrode and ST events in clinical practice over a period of 14 years (from 2000 to 2013) • Use of statistical tests (e.g., chi-squared and ANOVA linear trend analysis)	• The rate of acidemia was significantly reduced by 84% during the investigation period • Since a preceding RCT, which only assessed a limited number of years, reported a lower reduction in the rate of acidemia, two possible reasons for the obtained improvement were identified: (1) the introduction of the scalp electrode and ST events, and (2) the intensified training of the hospital personnel	• Most of the fetuses were monitored by using the scalp electrode, but no record was kept of which were monitored • Significant changes occurred in the population of fetuses over the years which were included in the study
Cagninelli et al. (2021) [59]	• Fetuses from six RCTs and seven meta-analyses of RCTs • Use of different definitions for acidemia (e.g., UA pH ≤ 7.05) • Use of intrapartum CTG tracings	• Review the effects associated with the use of the scalp electrode and ST events in clinical practice with regards to the onset of acidemia, the rate of operative deliveries as well as other perinatal outcomes	• Conflicting results were presented regarding potential benefits of using ST events to reduce the occurrence of acidemia in both the RCTs and meta-analyses of RCTs • This result could be motivated by the use of different definitions for acidemia in different studies	• Inclusion only of RCTs and seven meta-analyses of RCTs
Hulsenboom et al. (2022) [36]	• 20 total fetuses including the following: - 10 with acidemia (mean GA = 283 days, UA pH < 7.05) - 10 matched controls (mean GA = 278 days, UA pH > 7.20) • Use of CTG tracings from the last 10 h before delivery	• Comparison of the diagnostic accuracy of absolute and relative ST events with regard to acidemia • The relevance of absolute ST events was verified by two healthcare professionals who annotated CTG tracings • Use of a statistical test and metrics (i.e., McNemar’s test)	• Relative ST events returned higher sensitivity and specificity in predicting fetuses developing acidemia compared to absolute ST event (SE = 0.90 vs. 0.70, SP = 1.00 vs. 0.70) • No statistical differences were found between relative and absolute ST events	• Only a limited number of fetuses was included • A non-complete spectrum of UA pH values was considered to include fetuses
Tarvonen et al. (2024) [57]	• 213,798 fetuses (GA ≥ 37 weeks) including the following: - 81,559 monitored using Doppler ultrasound transducer (358 with UA pH < 7.0) - 62,268 monitored using both a Doppler ultrasound and a mHR transducer (139 with UA pH < 7.0) - 69,971 monitored using the fetal scalp electrode (130 with UA pH < 7.0) • Use of CTG tracings recorded during labor	• Investigation of the relationship between CTG tracings recorded during labor using different strategies with different fetal and neonatal complications • Use of statistical tests (e.g., Kruskal–Wallis test and Mann–Whitney U test)	• Fetuses monitored with the Doppler ultrasound transducer had a 2.2-fold increased risk of acidemia (defined as UA pH < 7.0) compared to those monitored with the other two strategies, a difference that remained significant after adjustment for maternal, delivery, and fetal risk factors • Fetuses monitored using the combination of a Doppler ultrasound transducer and a mHR transducer had a 1.4-fold increased risk of acidemia defined by composite criteria compared to those monitored using the scalp electrode	• Features such as ST events were not investigated within this study

**Figure A1:**
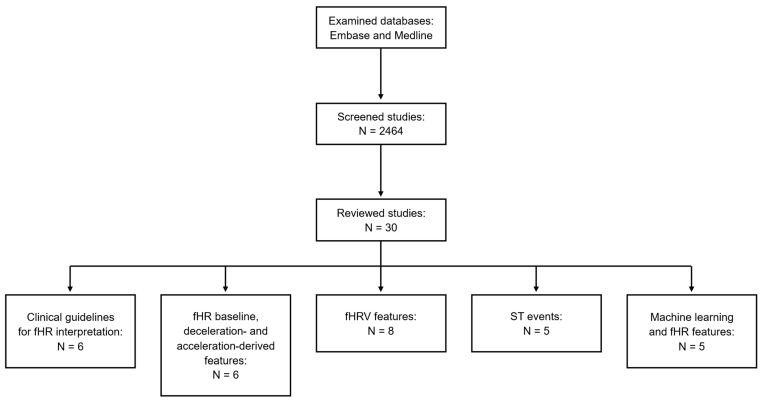
Screening process used to identify the studies to be included in this literature review.

## Data Availability

No new data were created or analyzed in this study.

## Abbreviations

The following abbreviations are used in this manuscript:

Abs	Absolute values (for the power spectral density computed in a spectral band)
AC	Acceleration capacity of the phase-rectified signal averaging
ACC	Accuracy
ACOG	American College of Obstetricians and Gynecologists
ANS	Autonomic nervous system
AUROC value	Area under the receiver operating characteristic curve value
BD	Base deficit
BE	Negative base excess
BiLSTM	Bidirectional long short-term memory network
CNN	Convolutional neural network
CTG	Cardiotocography
CTU-UHB dataset	Dataset collected by Czech Technical University in Prague and University Hospital in Brno
DC	Deceleration capacity of the phase-rectified signal averaging
DFFN	Deep feature fusion network
DR	Deceleration reserve of the phase-rectified signal averaging
FBS	Fetal scalp blood sampling
fECG	Fetal electrocardiogram
fHR	Fetal heart rate
fHRV	Fetal heart rate variability
FIGO	International Federation of Gynecology and Obstetrics
FSI	Fetal Stress Index
GA	Gestational age
HF	High-frequency range of power spectral density
IALS	Inverse average length of acceleration and deceleration segments
LF/HF ratio	Ratio between the power spectral density computed in the low-and high-frequency range
LF range	Low-frequency range of power spectral density
LTV	Long-term variability
LSTM	Long short-term memory networks
MF range	Mid- or movement frequency range of the power spectral density
mHR	Maternal heart rate
NICHD	National Institute of Child Health and Human Development
NPV	Negative predictive value
PNS	Parasympathetic nervous system
PIP	Percentage of inflection points
PRE	Precision
PRSA	Phase-rectified signal averaging
PSD	Power spectral density
PSS	Percentage of short accelerations or deceleration segments
QI	Quality index
Rel	Relative values (for the power spectral density computed in a spectral band)
RCT	Randomized controlled trials
ROC curve	Receiver operating characteristic curve
SE	Sensitivity
SMFM	Society for Maternal-Fetal Medicine
SNS	Sympathetic nervous system
SP	Specificity
STV	Short-term variability
SVM	Support vector machines
UA pH	Umbilical cord artery pH
UV pH	Umbilical cord vein pH
VLF range	Very low-frequency range of power spectral density

## Appendix A

### Screening Process and Inclusion Criteria

Two databases were screened using a systematic approach based on query blocks to search for studies to be included in this literature review. However, due to the very high number of studies that met the inclusion criteria, only a selection of studies was included in this literature review, which was therefore written using a narrative approach.

The first screening of the databases Embase and Medline was performed on 11 October 2023 by one author (I. H.) using three query blocks to include studies which met the following inclusion criteria: (#1) the use of fHR or fECG, (#2) a prediction task, and (#3) the investigation of acidemia. Query blocks included words or word combinations that were previously discussed and revised in multiple sessions by all authors and were specifically selected considering the indexing method that characterizes each database. In addition to this, specific words or word combinations were also searched in the title, abstract, and keywords of the studies included in each database. A fourth query block (#4) was then defined to find overlaps between the previously mentioned query blocks and to exclude animal or non-human studies as well as conference abstracts. As an example, query blocks that were used to search Embase are reported in Table A1. Identified studies from each database were then merged and deduplicated to avoid redundancy. A second and third screening of both databases were performed on 22 May 2024, and 24 November 2025, respectively, by the same author to check for new studies meeting the inclusion criteria. As a result of all screenings, a total of 2464 studies were identified as eligible for inclusion.

Two authors (G. V. and G. S.) performed a further screening to include studies following the separation presented in the Background section. This screening was performed by reading titles, abstracts, and keywords. Due to the very high number of studies that met the inclusion criteria for the different sections, a total of 30 studies were identified and reviewed in detail based on the year of publication (priority was given to studies published within the recent 5 years to increase awareness about their methods and results), the number of included fetuses, and the use of widely accepted clinical guidelines for fHR interpretation, fHR features, and ST events for acidemia prediction. Subsequently, the identified studies were separated based on the best-fitting section. The whole screening process therefore resulted in the final selection indicated in Figure A1.

**Table A1:** Query blocks used to perform literature search in Embase.

Query Block	Query Block Description	Words or Word Combinations Searched Within the Database Indexing, Titles, Abstracts and Keywords of the Studies
#1	Use of fHR and fECG	‘fetus electrocardiography’/exp OR ‘fetus heart rate’/exp OR ‘cardiotocography’/exp OR ((‘CTG*’ OR ‘cardiotoco*’ OR ‘electronic fetal monitor*’ OR ‘EFM*’):ti,ab,kw) OR (((fet* OR foet*) NEAR/3 (‘HR*’ OR ‘HRV’ OR ‘ecg*’ OR ‘ekg*’ OR ‘elektrocardiogra*’ OR ‘electrocardiogra*’ OR ‘cardiotocogra*’ OR ‘heart rate*’ OR ‘heart rate variabilit*’))):ti,ab,kw
#2	Prediction task	‘prediction and forecasting’/exp OR ‘statistical analysis’/exp OR ‘data classification’/exp OR ‘risk’/exp OR ‘machine learning’/exp OR ‘neural network’/exp OR ‘artificial intelligence’/exp OR (‘predict*’ OR ‘prognos*’ OR ‘correlat*’ OR ‘relevan*’ OR ‘statistic*’ OR ‘analys*’ OR ‘detect*’ OR ‘classific*’ OR ‘risk*’ OR ‘machine learning’ OR ‘deep learning’ OR ‘neural network*’ OR ‘artificial intelligence’):ti,ab,kw
#3	Investigation of acidemia	‘asphyxia’/exp OR ‘suffocation’/exp OR ‘fetus hypoxia’/exp OR ‘fetal acidosis’/exp OR ‘fetus acidemia’/exp OR ‘newborn hypoxia’/exp OR ‘fetus acid base balance’/exp OR ((fet* OR foet* OR perinat* OR neonat* OR newborn* OR premature* OR preterm* OR term*) NEAR/6 (asphyx* OR suffocat* OR hypox* OR acidem* OR acidos*)):ti,ab,kw
#4	Overlaps between query blocks and exclusion criteria	#1 AND #2 AND #3 NOT ([animals]/lim NOT [humans]/lim) NOT ‘conference abstract’/it
